# Liquid biopsies for detection, characterization, and interception of therapy resistance in thoracic malignancies

**DOI:** 10.3389/fonc.2026.1806868

**Published:** 2026-08-10

**Authors:** Ezgi Oner, Volga M. Saini, Christina Cahill, Hannah O’Toole, Jennifer W. Li, Kathy Gately, Valsamo Anagnostou

**Affiliations:** 1 The Sidney Kimmel Comprehensive Cancer Center, Johns Hopkins University School of Medicine, Baltimore, MD, United States; 2 Thoracic Oncology Research Group, Trinity Translational Medicine Institute, St James’s Hospital, Dublin, Ireland; 3 Department of Clinical Medicine, School of Medicine, Trinity College Dublin, Dublin, Ireland; 4 Trinity St. James’s Cancer Institute, Trinity College Dublin, Dublin, Ireland; 5 The Lung Cancer Precision Medicine Center of Excellence, Johns Hopkins University School of Medicine, Baltimore, MD, United States; 6 The Johns Hopkins Molecular Tumor Board, Johns Hopkins Hospital, Baltimore, MD, United States

**Keywords:** circulating tumor cells (CTCs), circulating tumor DNA (ctDNA), liquid biopsies, lung cancer, therapy resistance, thoracic malignancies

## Abstract

Therapy resistance remains a leading cause of treatment failure and mortality in thoracic malignancies, despite major advances in targeted therapies and immunotherapies. Resistance evolves through heterogeneous, patient-specific mechanisms, including genetic alterations, epigenetic reprogramming, lineage plasticity, and tumor-microenvironment interactions, often emerging before radiographic progression or clinical relapse. Conventional tissue biopsies and imaging, while essential for diagnosis and treatment selection, are limited in their ability to capture spatial and temporal tumor heterogeneity or to support dynamic monitoring of tumor evolution. Liquid biopsy has therefore emerged as a minimally invasive approach for real-time assessment of tumor-derived biomarkers in circulation, enabling longitudinal tracking of resistance biology across the cancer care continuum. In this review, we highlight recent advances in liquid biopsy applications for thoracic malignancies, focusing on circulating tumor DNA (ctDNA) and circulating tumor cells (CTCs) as complementary analytes for baseline molecular profiling, detection of primary and acquired resistance to therapy, and identification of minimal residual disease and molecular relapse. Beyond mutation-based approaches, we highlight emerging non-genomic ctDNA features, including epigenomic and fragmentomic signatures, that capture treatment-induced adaptation and lineage plasticity not detectable by conventional plasma genomic profiling, and discuss advances in CTC technologies that preserve cellular and phenotypic context relevant to resistance and metastatic potential. Finally, we examine multimodal liquid biopsy strategies that integrate multiple circulating analytes with artificial intelligence-assisted methods to enhance sensitivity, provide a more comprehensive view of tumor biology, and inform adaptive therapy strategies. We also outline key analytical and clinical challenges that must be addressed through standardized, prospective trials to translate liquid biopsy-guided surveillance and early interception of resistance into improved patient outcomes.

## 1 Introduction

Thoracic malignancies remain the leading cause of cancer-related mortality among both males and females worldwide (1). Despite substantial advances in standard-of-care treatment, including immunotherapy and targeted therapies, a significant subset of patients ultimately develops therapeutic resistance and experiences disease recurrence. Resistance may arise from the outgrowth of pre-existing treatment-resistant clones during therapy (primary or intrinsic resistance) or from the persistence and subsequent expansion of drug-tolerant cells following an initial response (secondary or acquired resistance) (2). These resistance mechanisms are multifactorial, highly patient-specific, and even heterogeneous within individual tumors. They may be driven by genomic instability, epigenetic alterations, tumor microenvironment–related factors, or pharmacokinetic treatment failure (2–5). Consequently, there remains a critical unmet need to better understand the biology of resistance and, equally importantly, to enable effective disease monitoring for early detection, characterization, and timely interception of therapy resistance.

Tissue biopsy and radiographic imaging currently constitute the clinical standard for initial diagnosis and staging, assessing patient eligibility for immunotherapy and targeted therapies through tumor profiling, and monitoring treatment response and disease progression. However, tissue biopsy is constrained by its invasiveness, limited accessibility for tumors in difficult-to-reach locations, inability to support frequent repeated sampling, and restricted representation of intratumoral heterogeneity because it samples only a small part of the tumor. Similarly, radiographic imaging lacks sufficient sensitivity to detect minimal residual disease (MRD) that may ultimately drive relapse. To address these limitations, liquid biopsy has emerged as a minimally invasive, real-time approach to cancer detection and monitoring through analysis of tumor-derived biomarkers in biological fluids, most commonly blood, but also in pleural effusions, saliva, urine, and exhaled breath. Unlike traditional tissue biopsy, which requires surgical or needle-based tumor sampling, a simple blood draw captures tumor-derived components actively shed into the circulation, including circulating tumor DNA (ctDNA), circulating tumor cells (CTCs), extracellular vesicles (EVs), and tumor-associated proteins (6, 7).

Among the various liquid biopsy analytes, ctDNA and CTCs are the most extensively investigated. ctDNA is the tumor-derived fraction of circulating cell-free DNA (cfDNA) released into the bloodstream, primarily through tumor cell apoptosis and necrosis, and reflects the genomic and epigenomic profiles of the originating tumor (8). CTCs are tumor cells that have detached from primary or metastatic lesions and entered the bloodstream, providing rich phenotypic and molecular information about their tumor of origin (9). Although the low abundance of ctDNA and CTCs, along with the cost and sensitivity limitations of detection technologies, have historically limited their clinical implementation, recent advances in next-generation sequencing (NGS) and artificial intelligence have enabled broader application for longitudinal disease monitoring and treatment guidance (10). Liquid biopsies can be applied across the cancer care continuum, from initial molecular profiling to longitudinal disease surveillance for progression, recurrence, and acquired resistance (11–14). By enabling real-time assessment of ctDNA, CTCs, and other circulating analytes, liquid biopsies provide dynamic insight into tumor genomics and clonal evolution under selective therapeutic pressure, thereby complementing information from tissue-based biopsies.

Overall, liquid biopsies are particularly advantageous when tissue is unavailable or when repeat biopsies are impractical, enabling dynamic longitudinal monitoring through serial sampling. It can also deliver faster results than tissue biopsy—especially when urgent treatment decisions are required—while providing a more comprehensive snapshot of tumor heterogeneity by capturing genetic alterations originating from multiple tumor sites. However, there are still limitations, including sensitivity issues, especially in early-stage or low-burden disease, where CTC counts or ctDNA levels may be too low to detect. As with a tissue biopsy, a “negative” liquid biopsy does not always mean there is no mutation present. Selecting a liquid biopsy technology involves a critical trade-off between the depth of known targets and the breadth of genomic discovery. Targeted assays provide high sensitivity for characterized mutations, but they might miss *de novo* resistance or rare driver mutations; conversely, broader panels often lack the sensitivity required to detect rare mutations in early-stage disease. Other issues include cost and access, as advanced NGS-based liquid biopsy tests can be expensive and may not be widely available in all healthcare systems.

## 2 ctDNA-based assays

### 2.1 Technologies for ctDNA detection and characterization

**Figure 1:**
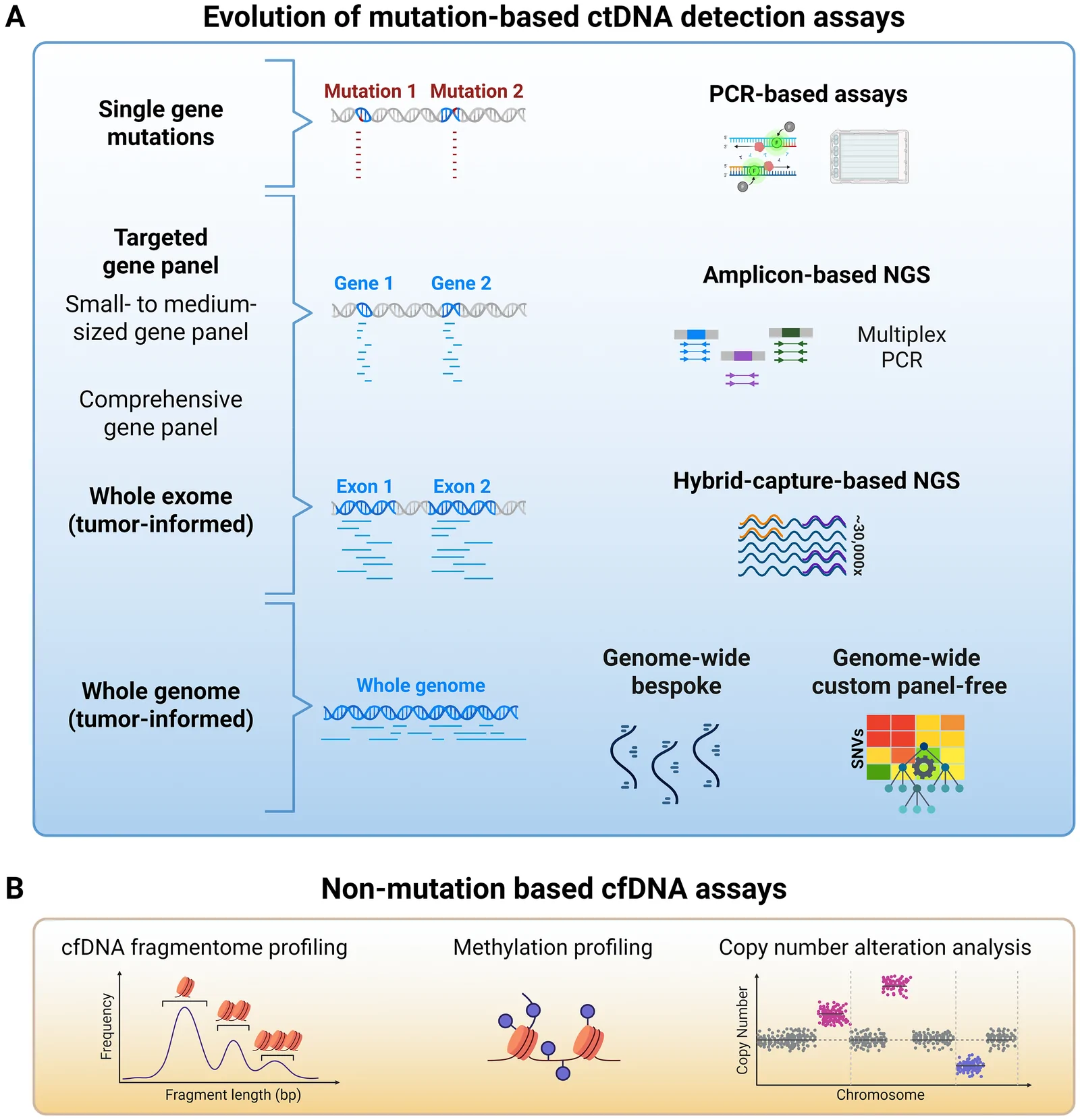
Overview of circulating tumor DNA (ctDNA) detection technologies. **(A)** Clinically validated mutation-based ctDNA detection techniques have evolved from polymerase chain reaction (PCR) to next-generation sequencing (NGS), expanding from single-gene mutation detection using real-time PCR to broader hybrid-capture gene panels, and ultimately to coding-region analysis using whole-exome sequencing (WES) and genome-wide profiling using whole-genome sequencing (WGS). Across these technologies, statistical and machine-learning models may be applied to enhance error correction, variant interpretation and clinical reporting, particularly for WES/WGS data. **(B)** Beyond mutation-based ctDNA assays, cfDNA can capture fragmentomic, epigenomic, and structural genomic features through targeted methylation assays or genome-wide approaches integrated with machine learning methods. cfDNA, cell-free DNA; ctDNA, circulating tumor DNA; PCR, polymerase chain reaction; NGS, next-generation sequencing; WES, whole-exome sequencing; WGS, whole-genome sequencing. Created in https://BioRender.com.

ctDNA-based approaches can provide information on genomic alterations—including single-nucleotide variants (SNVs), indels, copy-number alterations (CNAs), and structural variants such as fusions—as well as non-genomic cfDNA features. The latter include topology, epigenomic characteristics, such as DNA methylation, chromatin organization, histone modifications, and transcription factor binding site accessibility, as well as fragmentomic patterns reflecting nucleosome positioning, fragment size, and fragment end profiles (15). Although non-genomic approaches have advanced rapidly, clinically validated ctDNA assays are predominantly genomic-based (16). ctDNA assays can be categorized by whether a tissue sample is required as tumor-naïve or tumor-informed approaches, with the latter leveraging genomic and/or epigenetic data from tumor tissue DNA. These methods may also require matched white blood cell (WBC)-derived DNA to eliminate biological noise from clonal hematopoiesis (CH) (17). **Figure 1** provides an overview of ctDNA detection techniques.

Optimal ctDNA assay selection is based on analytical and clinical sensitivity and specificity, considering the intended use and diagnostic purpose (8, 18). The limit of detection (LoD) for tumor-naïve mutation-based ctDNA assays generally ranges from 0.01% to 0.1% variant allele frequency (VAF) (1 in 10,000 to 1 in 1,000), such as polymerase chain reaction (PCR)-based assays (19, 20) or targeted NGS integrated with error-correction algorithms (21–23). In contrast, bespoke tumor-informed mutation-based ctDNA assays display lower LoD values (generally 0.01% to 0.001%, and as low as 0.0001%), however may be limited by feasibility challenges related to tissue accessibility and purity, high costs, and long turnaround times associated with the customized approach (24–27). Although most clinically available ctDNA assays are targeted gene panels, whole-genome sequencing (WGS) approaches are emerging to provide a global, unbiased genomic profile, enabling detection of genome-wide CNAs, structural variants, and mutational signatures (28). WGS of cfDNA, matched tumor, and WBC samples can achieve an LoD of 0.001% tumor fraction (TF) when combined with an error correction method (29), and may reach as low as 0.0001% with deep learning-guided signal-to-noise ratio enhancement (30).

While less sensitive for ultra-low-frequency variant detection, tumor-naïve whole genome-based approaches leverage genome-wide cfDNA signals to capture the architecture and biology of cfDNA, enabling both genomic and epigenomic characterization (31). Genome-wide fragmentation profiling using low-coverage WGS combined with machine learning enables early lung cancer detection, as demonstrated by DELFI (32, 33) and its clinically validated version, FirstLook Lung (34). Machine learning-integrated, tumor-naïve whole-genome mutational signature approaches, such as GEMINI for lung cancer, were developed primarily for noninvasive cancer detection; however, pilot data also support their application to monitor TKI therapy response and disease progression in advanced non-small cell lung cancer (NSCLC) (35). These mutation- and non-mutation-based ctDNA assays are introduced below, and details of the selected studies investigating their utility in the context of therapy resistance in thoracic malignancies are provided in **Supplementary Table 1**.

#### 2.1.1 Tumor-naïve mutation-based ctDNA assays

In the context of therapy resistance, the first clinical applications of liquid biopsy were for the selection of second-line treatment based on the detection of acquired resistance mutations using real-time PCR, representing the earliest Food and Drug Administration (FDA)-approved ctDNA tests, such as the Cobas^®^ *EGFR* Mutation Test v2 and the Therascreen^®^ *PIK3CA* Mutation Kit (36, 37). Although these PCR-based methods are well established, easy to perform, and cost- and time-efficient—and sensitivity for ctDNA quantification has been improved by up to 100-fold with digital PCR approaches, including microfluidic droplet digital PCR (ddPCR) and Beads, Emulsions, Amplification, and Magnetics (BEAMing)—they remain limited to the detection of one or a small number of pre-defined mutations and lack high multiplexing capacity (8, 16).

Advances in targeted NGS technologies, using amplicon- or hybrid-capture-based enrichment, have enabled massively parallel sequencing of broader panels of genomic alterations and now constitute the vast majority of tumor-naïve ctDNA assays (8, 16). Amplicon-based multiplex PCR approaches use primers and employ predefined gene panels designed for pan-cancer applications or tailored to specific cancer (sub)types, with Oncomine™ Pan-Cancer (38), Oncomine™ Lung (39), and InVisionFirst-Lung (40) as representative examples. Hybrid-capture panels use biotinylated probes to target a fixed set of genes independent of the patient’s tumor and range from medium-sized panels to large comprehensive genomic profiling (CGP) assays covering hundreds of genes and multiple variant classes (SNVs, indels, CNAs, and gene fusions) (8). These tumor-naïve approaches—including the FDA-approved Guardant360 CDx (41), FoundationOne Liquid CDx (42) and Agilent Resolution ctDx FIRST plasma assay (43), as well as other assays such as PGDx elio™ plasma resolve (44) and the AVENIO ctDNA Surveillance kit (45)—are widely adopted for plasma CGP in the metastatic setting, particularly when tumor tissue is unavailable, due to their relatively high sensitivity and broad genomic coverage. Notably, MSK-ACCESS demonstrated high concordance with FoundationOne Liquid CDx in VAF detection, with a concordance correlation coefficient of 0.98 (Pearson’s r=0.98, P<0.001) (46). Targeted error-correction sequencing (TEC-Seq) had an LoD of 0.05% and identified molecular responders with improved survival in NSCLC (23, 47, 48), small-cell lung cancer (SCLC) (49), and esophageal cancer (23, 50). Cancer Personalized Profiling by Deep Sequencing (CAPP-Seq) integrated with digital error suppression (IDES), improved LoD from 0.02% to 0.0025% compared with the original CAPP-Seq approach using prior tumor tissue mutation data (22, 51).

Whole exome sequencing (WES)-based cfDNA sequencing relies on hybrid capture-based enrichment using exon-targeting probes to selectively sequence coding regions of the genome. Although both WES and many CGP assays can employ hybrid capture, they differ fundamentally in genomic breadth, achievable sequencing depth, and analytical sensitivity, and in their typical use—WES for comprehensive variant discovery in exploratory studies (52–56), and CGP for higher−depth, clinically focused detection of actionable alterations. In a recent molecular tumor board report, WES revealed additional actionable alterations compared with targeted sequencing (up to 203 genes), but only a small subset of the 38 patients with advanced cancer experienced added clinical benefit (57). Tumor−naïve WES captures the coding mutational landscape but is limited in sensitivity for low−ctDNA samples; tumor−informed or targeted approaches are generally better suited for detecting low−frequency variants. To increase the effective depth of WES without additional cost, one strategy involves initially applying low-coverage WGS to estimate cfDNA TF in all samples, followed by WES only in selected samples with TF above a predefined threshold, while directing samples with lower TF to targeted sequencing (53).

#### 2.1.2 Tumor-informed mutation-based ctDNA assays

Tumor-informed assays provide superior analytical sensitivity and specificity but increase invasiveness, risk of assay failure when tissue samples have low tumor purity, cost, and processing time (58). Tumor-informed bespoke ctDNA assays are individually designed for each patient and offer ultra-high sensitivity, making them particularly well suited for MRD detection in early-stage disease. These approaches typically begin with mutation profiling of tumor tissue by WES, followed by matched WBC sequencing to exclude germline variants (8). Early implementations relied on multiplex PCR (amplicon)-based enrichment strategies and tracked a limited number of patient-specific somatic variants, as exemplified by Signatera™ (16 variants) (24) and RaDaR (48 variants) (25). Subsequent methodological advancements have increased sensitivity through different strategies such as optimization of amplicon-based enrichment and expansion of target breadth via hybrid-capture-based sequencing. For instance, the anchored multiplex PCR-based personalized assay ArcherDx leverages a gene-specific primer on only one end of the target and a universal adapter on the other, enabling robust amplification from low-input and fragmented cfDNA (59, 60). Their key advantage lies in tailoring assay design to each individual’s mutation profile; however, they are generally limited by tracking up to ~50 somatic variants across multiple genes, thereby restricting genomic coverage compared with genome-wide approaches. Hybrid-capture-based approaches have enabled broader mutation tracking by capturing hundreds to thousands of patient-specific variants. Expanding the target size of custom panels based on matched tumor WGS has achieved 1–3 parts-per-million (ppm) sensitivity at 99.9% specificity with NeXT Personal (26), whereas the LoD of PhasED-Seq reached ~1 ppm using a panel covering personalized phased variants identified from tumor tissue WGS (27). When cfDNA WGS is combined with tumor-informed guidance, as in MRDetect (29), MRD-EDGE (30), and Labcorp PlasmaDetect (61), it may achieve the ultra-high sensitivity required to detect low tumor shedding, particularly for MRD in early-stage cancers. Recent advances in machine learning and their integration into WGS-based tumor-informed platforms can detect low-shedding tumors such as diffuse pleural mesothelioma, where radiographic response assessment is often inadequate (18, 61). An illustrative example is the signal-to-noise enrichment strategy implemented in the machine learning-guided WGS ctDNA SNV and copy number variation (CNV) platform MRD-EDGE, which improved post-operative MRD detection in resectable NSCLC and colorectal cancer and enabled more accurate longitudinal tracking of ctDNA dynamics during neoadjuvant ICI therapy with or without stereotactic body radiotherapy (SBRT) in early-stage NSCLC, outperforming the earlier sequencing error-suppression WGS method MRDetect (30). A recent tumor-informed cfDNA WGS approach, called AccuScan, achieved a single-read-level error correction rate of 4.2 × 10^-7^ through rolling circle amplification (concatemer sequencing) combined with repeat confirmation, reaching ≤ 10 ppm sensitivity at ~60x coverage for MRD detection in colorectal and esophageal cancers and for monitoring immunotherapy response in melanoma (62).

#### 2.1.3 Non-mutation-based ctDNA assays

Characterization of circulating cfDNA through epigenomic and fragmentomic analyses has emerged as a powerful complement to mutation-based ctDNA assays; enabling detection of MRD after curative-intent surgery or definitive therapy (63, 64), dynamic identification of early molecular progression preceding radiographic imaging (63, 65, 66), characterization of therapy-associated shifts in tumor cellular composition or tissue-of-origin signals linked to resistance (67), and enhanced sensitivity for detecting recurrent or resistant disease when integrated with mutation-based approaches (64, 68) in thoracic malignancies (**Supplementary Table 1**). These studies have employed targeted methylation panels, genome−wide bisulfite or enrichment−based methylome sequencing, and fragmentomics approaches; many combine non-mutation-based features with machine learning classifiers to maximize sensitivity and specificity for detection and longitudinal monitoring.

Methylation-based epigenomic ctDNA profiling can elucidate treatment resistance, as DNA methylation is an early, tissue- and tumor type-specific event in carcinogenesis, enabling deep longitudinal assessment of CpG island and other differentially methylated region (DMR) alterations, and facilitating MRD detection, early progression tracking despite intratumoral heterogeneity, and stratification of recurrence risk and response to chemotherapy, targeted therapy, and immunotherapy (69). In ctDNA methylation profiling, targeted panels typically employ hybrid-capture enrichment following bisulfite conversion (ELSA-seq (70)), whereas genome-wide detection of methylated cytosines relies on either bisulfite-based whole-genome bisulfite sequencing (WGBS) or enzymatic conversion-based sequencing (EM-seq (71, 72)). The tumor-naïve Guardant Reveal assay enriches methylated cfDNA via bead-based methylation binding domain affinity, applies methylation-specific enzymatic restriction, and analyzes >20,000 regions that distinguish cancer-derived from healthy cfDNA (73). Dynamic monitoring of chemotherapy response in advanced solid tumors, including lung cancer, using methylated cfDNA TF analyses by the Guardant Reveal, demonstrated that patients with a ≥98% TF decrease or continuous TF decline had superior real-world outcomes, and that TF increases preceded initiation of the next line of therapy by a median of 2.27 months (74). Galleri™ initially implemented WGBS but was later refined to a targeted methylation panel covering >100,000 regions (75). Tumor-naïve, whole-genome-based methylation profiling can use WGBS to sequence bisulfite-converted cfDNA, enabling integrated analyses of differentially methylated regions along with fragmentomics and CNV features, as in Expanded Multi-Modal Analysis (EMMA) (76). In contrast, antibody-based enrichment methods, such as cell-free methylated DNA immunoprecipitation and high-throughput sequencing (cfMeDIP-seq) (77), capture genome-wide enriched methylated cfDNA regions using a 5-methylcytosine antibody and do not require bisulfite or enzymatic conversion.

cfDNA fragmentomics leverage tumor-independent patterns in fragment size, jagged ends, and fragment-end motifs to estimate tumor burden without prior knowledge of mutations. Most fragmentomic approaches were initially developed for early lung cancer detection; however, a recent study demonstrated that DELFI-tumor fraction (DELFI-TF) machine-learning method using low-coverage WGS produces cfDNA TF scores that strongly correlate with maximum mutant allele frequency values (maxMAFs) from a targeted panel NGS assay (r=0.93, p<0.0001), predict treatment response, and independently estimate overall survival (OS) in metastatic lung and colorectal cancer (78). Similarly, the Fragle deep-learning model, which quantifies ctDNA levels as high (>1%) or low (≤1%) based on cfDNA fragment length density distributions, achieved 94% detection rate at an LoD of ~1% and has been proposed as a complementary approach to targeted panel NGS to more accurately determine treatment response and detect MRD (79).

While cfDNA tumor-naïve, non-genomic approaches show promise in advanced cancers, their sensitivity for MRD detection in early-stage NSCLC remains limited, requiring tissue-informed strategies combined with mutation profiles (64) or machine-learning classifiers (63). Recent advances offer promise, as demonstrated by Moldovan et al. (80): a tumor-naïve, multi-modal approach combining cfDNA fragmentomics (fragment-end composition and size) with genomic TF detected 72% of cancers across multiple types at 95% specificity and was associated with poorer survival in resectable esophageal adenocarcinoma and lung cancer, highlighting its potential for recurrence prediction.

### 2.2 Applications of ctDNA-based assays

#### 2.2.1 Re-genotyping via ctDNA-based liquid biopsies identifies actionable drivers of acquired resistance to targeted therapies

Targeted therapies have improved outcomes for patients with oncogene-driven thoracic cancers; however, acquired resistance is common (81, 82). ctDNA-based liquid biopsies play an important role in identifying resistance mechanisms, which may involve on-target mutations, bypass signaling activation, or histologic transformation. Clinically, ctDNA assays are often preferred at baseline to guide the use of targeted therapies when actionable genomic alterations are identified (11, 12, 14), or at progression, particularly when repeat tissue biopsies are not feasible or undesirable. Furthermore, they may capture concurrent resistance alterations that tissue sampling bias can miss, providing a more comprehensive picture of tumor evolution (83).

Using EGFR-mutant NSCLC as an example, EGFR tyrosine kinase inhibitors (TKIs) have been used across disease settings, including neoadjuvant and adjuvant settings for localized disease and as treatment alone or in combination with chemotherapy for metastatic disease (84–88). Despite durable responses, multiple on-target and off-target resistance mechanisms can emerge (89, 90). Liquid biopsy can identify secondary *EGFR* mutations (e.g. C797S), *MET* alterations, *ERBB2* amplifications, *PIK3CA* mutations, and other bypass signaling alterations with direct therapeutic implications. For example, detection of *MET* amplifications or exon 14 skipping alterations through ctDNA analyses has facilitated the rational use of combination strategies with EGFR TKIs and MET inhibitors (91, 92). Although current guidelines recommend re-biopsy of tissue at progression to guide new treatment strategies against identified resistance mechanisms (81, 93), this may not always be feasible in practice, as observed in 61% of 154 EGFR-mutant NSCLC patients (94).

Acquired resistance mechanisms to EGFR TKIs have been also widely investigated through cfDNA methylation either alone (95) or in combination with mutation-based assays (68, 96). Studies employing parallel cfDNA mutation and methylation profiling have demonstrated the complementary value of epigenomic information for monitoring response and resistance in EGFR-mutant NSCLC. Nguyen et al. showed that distinct EGFR-TKI resistance mechanisms revealed distinct methylation landscapes, with EGFR amplification associated with genome-wide hypomethylation, genomic instability, and treatment response duration, whereas MET and HER2 amplification lacked comparable methylation changes (96). Consistently, Xia et al. reported that ctDNA methylation levels tracked closely with mutational burden during therapy, with coordinated decreases in methylation and maximum allele fraction reflecting treatment efficacy and subsequent increases preceding clinical disease progression (68). Similarly, in ALK-mutant NSCLC, longitudinal cfDNA tumor-specific 5-methylcytosine (5-mC) scores generated using bisulfite-free cfMeDIP-seq were highly correlated with cfDNA genomic alterations, associated with poorer survival, and increased prior to radiographic disease progression, demonstrating utility for monitoring resistance to ALK TKIs (66). Beyond combined genomic and methylation approaches, integrating multiple epigenomic features effectively informs TKI resistance. For example, histologic transformation to SCLC is an underdiagnosed mechanism of TKI resistance in EGFR-mutant lung adenocarcinoma, but epigenomic cfDNA profiling integrating histone modifications, DNA methylation, and chromatin accessibility can accurately detect small-cell transformation, achieving high predictive performance (AUC 0.94) and enabling non-invasive discrimination from adenocarcinoma using just 1 mL of plasma (67).

All four FDA-approved ctDNA tests for NSCLC are tumor-naïve, using genetic characterization of ctDNA to identify actionable driver mutations, and they are used to manage treatment in the metastatic setting (i.e., initial selection of targeted therapy and monitoring treatment response and resistance) (97). Although the first FDA-approved assay was the RT-PCR-based cobas^®^ *EGFR* Mutation Test v2, recent advances in NGS have led to the rapid and steady adoption of hybrid-capture-based CGP assays—including Guardant360 CDx, FoundationOne Liquid CDx, and the Agilent Resolution ctDx FIRST assay—in advanced NSCLC clinics. Cobas^®^ *EGFR* plasma testing demonstrated 97.9% specificity and 72.1% sensitivity in the ENSURE, FASTACT-2, and ASPIRATION trials (98), and its longitudinal use enabled early detection of emerging *EGFR* T790M mutations to guide timely switching from first- to third-generation TKIs, improving progression-free survival (PFS) in the APPLE trial (37). Guardant360 CDx, a 74-gene panel, is FDA-approved plasma CGP to detect selected alterations in *EGFR* (exon 19 deletions, exon 20 insertions, exon 21 L858R, and T790M), *KRAS* G12C, and *ERBB2* for TKI treatment management in advanced NSCLC, as well as *ESR1* for breast cancer.

In the FLAURA and AURA3 trials conducted among patients with EGFR-mutated advanced NSCLC, plasma ctDNA NGS with Guardant360 CDx enabled detection of acquired resistance to osimertinib at progression, with first-line cases showing no *EGFR* T790M-mediated resistance, while second-line cases showing T790M loss in ~50%; both cohorts displayed recurrent *MET* amplification (16–18%) and *EGFR* C797 alterations (6–18%) (99, 100). Guardant360 CDx also showed strong analytical performance (LoD 0.1–0.5%, 98–100% specificity) and clinical validity, yielding PFS benefits consistent with tissue-based selection for osimertinib (101) and supporting *KRAS* G12C detection for sotorasib, with acceptable tissue concordance (41). Likewise, although FDA-approved only to identify *KRAS* G12C-mutant NSCLC patients eligible for adagrasib, the Agilent Resolution ctDx FIRST assay was associated with improved survival in patients receiving ctDNA-matched targeted therapies and revealed subclonal resistance drivers (e.g., *PIK3CA* and *RICTOR* alterations) missed by tissue profiling, demonstrating independent prognostic value in advanced NSCLC (46). Moreover, FoundationOne Liquid CDx is a 324-gene plasma CGP assay with FDA approval for detecting actionable alterations in NSCLC (*EGFR* exon 19 deletion or exon 21 L858R, *ALK* and *ROS1* rearrangements, *MET* exon 14 skipping variants/indels, *BRAF* V600E), *NTRK1*/2/3 fusions in solid tumors, and selected indications in other cancers (*BRCA1/BRCA2/ATM/BRAF* in metastatic castration-resistant prostate cancer and *PIK3CA* in breast cancer). In the PRISM study, guided by a weekly molecular tumor board, paired FoundationOne Liquid CDx and tissue CGP (FoundationOne CDx) demonstrated faster turnaround and fewer testing failures with ctDNA, and improved detection of therapy-driven tumor evolution (higher detection of mutations and rearrangements and growing discordance with archival tissue over time), but lower sensitivity for CNVs, supporting its complementary or alternative use for guiding treatment decisions in advanced solid tumors, including NSCLC (102).

In addition to FDA−approved ctDNA assays, an early proof−of−principle study showed that serial exome sequencing of plasma ctDNA can non-invasively track clonal evolution and emergent resistance—complementing tissue biopsies by identifying resistance mutations as they arise, for example the emergence of *EGFR* T790M after TKI therapy in an advanced NSCLC case (52). These findings, together with subsequent clinical studies, support the role of plasma ctDNA in capturing the genomic landscape of primary resistance, clonal evolution, and the emergence of acquired resistance to targeted therapies. Beyond guiding targeted therapy selection and response/resistance monitoring, dynamic ctDNA-guided (de)-escalation strategies enabled prolonged TKI breaks without compromising PFS in advanced NSCLC (103).

Prospective studies further highlight the clinical utility of ctDNA-guided resistance assessment through longitudinal ctDNA monitoring and adaptive treatment strategies (**Table 1**). NCT07058519 is an interventional trial based on ctDNA monitoring in locally advanced or metastatic *EGFR*-mutant NSCLC, with acquired resistance mechanisms assessed at progression. Similarly, RESISTYR (NCT05020275) aims to identify biomarkers of drug response and identify mechanisms of resistance to osimertinib. Even if no actionable alterations are identified, liquid biopsy results can still guide clinical decisions by supporting adjustments to systemic therapy or options for clinical trial enrollment.

**Table 1:** Representative ctDNA observational and interventional clinical trials in thoracic malignancies.

NCT	Study type	Setting/disease	Estimated enrollment	Assay/analyte, if specified	Study endpoints
NCT06105177	Observational	At diagnosis Lung	800	CTCs, ctDNA	Assess feasibility of incorporating upfront ctDNA analysis into clinical care
NCT05704530	Interventional	Resectable Esophageal	248	ctDNA	Characterize MRD dynamics during adjuvant immunotherapy and association of status with disease recurrence
NCT05254782	Observational	Early-stage, resectable NSCLC	360	ctDNA	Evaluate ctDNA detection rates and their association with disease relapse or recurrence
NCT04153526	Observational	Stage I-IIIB NSCLC	100	ctDNA	ctDNA detection rate
NCT05921474	Observational	Stage I-IIA NSCLC	100	ctDNA	Determine whether MRD predicts disease recurrence after SABR
NCT05167604	Observational	Stage IB-IIA NSCLC	150	ctDNA	3-year DFS
NCT06081777	Observational	Stage III NSCLC	65	ctDNA	2-year DFS
NCT06198868	Observational	Stage IIA-IIIC NSCLC	60	CTCs, ctDNA	PFS
NCT04976296	Observational	Stage I-IIIA, resectable NSCLC	300	ctDNA	Evaluate association of MRD with survival outcomes
NCT05965024	Observational	Stage IB – IIIB NSCLC	377	ctDNA	Two-year recurrence-free survival rate
NCT03791034	Observational	Stage I – IIIA NSCLC	700	ctDNA	Incidence of primary tumor recurrence
NCT05536505	Interventional - Phase 2	Stage IB-IIIB, resectable mEGFR NSCLC	180	ctDNA	Compare 3-year DFS rates of MRD positivity and MRD negativity postop
NCT04841811	Interventional - Phase 3	Stage III, unresectable mEGFR NSCLC	192	ctDNA	Objective response rate to almonertinib and 18-month event-free survival
NCT04966663	Interventional - Phase 2	Early stage, resectable NSCLC	66	ctDNA	Relapse-free survival
NCT04585477	Interventional - Phase 2	Stage IA2 - IIIC or locoregionally recurrent NSCLC	80	ctDNA	Characterize MRD dynamics following durvalumab
NCT04585490	Interventional - Phase 3	Locally advanced, unresectable NSCLC	48	ctDNA	Characterize MRD dynamics following chemotherapy
NCT05286957	Interventional - Phase 2	Stage IIA-IIIB, resectable NSCLC	60	ctDNA	Two-year PFS
NCT04093167	Interventional - Phase 2, 3	Metastatic NSCLC	230	ctDNA	Assess concordance between molecular response and radiologic response; Three-year PFS and OS
NCT07058519	Interventional - Phase 2	Metastatic mEGFR NSCLC	250	ctDNA	PFS and time to first ctDNA mEGFR relapse
NCT05020275	Observational	Metastatic mEGFR NSCLC	60	ctDNA	Evaluate relationship between plasma exposure to osimertinib and treatment response
NCT06167460	Observational	Locally advanced or metastatic NSCLC	50	ctDNA	Evaluate relationship between ctDNA dynamics and TKI response and survival outcomes
NCT06234579	Observational	Locally advanced or metastatic ALK+ NSCLC	108	ctDNA	Determine the proportion of patients with available NGS at diagnosis and retesting at progression
NCT06717243	Observational	ES-SCLC or advanced LCNEC	111	CTCs, ctDNA	Identification of alterations associated with chemo-immunotherapy resistance
NCT05257551	Observational	ES-SCLC	50	ctDNA	Determine whether transcriptional subtypes can be detected by Tempus assays; and Evaluate the association of subtype with clinical outcomes
NCT06227728	Observational	Metastatic Solid tumors, including lung and esophageal	50	ctDNA	Evaluate relationship between ctDNA dynamics and clinical response
NCT05585684	Observational	Metastatic, or imminent progression Solid tumors, including lung and esophageal	150	ctDNA	Assess prevalence of ctDNA variants; Determine the proportion of patients receiving molecular tumor board (MTB)-guided treatment; and Evaluate turnaround times from liquid biopsy collection to MTB recommendation and treatment initiation

CTCs, circulating tumor cells; ctDNA, circulating tumor DNA; mEGFR, mutated epidermal growth factor receptor; NSCLC, non–small cell lung cancer; DFS, disease-free survival; PFS, progression-free survival; TKI, tyrosine kinase inhibitor; 1L, first-line treatment; 2L, second-line treatment; ALK, anaplastic lymphoma kinase; ChemoIO, chemotherapy plus immunotherapy; ES-SCLC, extensive-stage small cell lung cancer; LCNEC, large cell neuroendocrine carcinoma; SCLC, small cell lung cancer; PD-L1, programmed death-ligand 1; MRD, minimal residual disease; SABR, stereotactic ablative radiotherapy; MTB, molecular tumor board; NGS, next-generation sequencing.

#### 2.2.2 Clinical utility of ctDNA-based liquid biopsies in detecting primary and acquired immunotherapy resistance

Immunotherapy has transformed the treatment landscape for advanced NSCLC without actionable genomic alterations, but only a small subset of patients benefits. Patients may exhibit primary resistance, defined as progressive or stable disease lasting less than 6 months, while others develop acquired resistance after an initial response of at least 12–24 weeks (104). Currently, programmed death-ligand 1 tumor proportion score (PD-L1 TPS) is the only clinically approved biomarker used to guide immune checkpoint inhibitor (ICI) selection in NSCLC, whereas tumor mutational burden (TMB) has limited use in routine clinical practice, largely due to a lack of standardization in testing methods and threshold values. A similar limitation applies to PD-L1 TPS, particularly in the 1–49% range, where optimal cut-off values to guide the choice between ICI monotherapy and combination with chemotherapy remain less well-defined. Moreover, its prognostic value is uncertain, as it may not fully capture intratumoral heterogeneity due to variability related to the biopsy site and its nature as a single time-point “snapshot” of tumor biology (105). Therefore, liquid biopsies, particularly the utilization of ctDNA, can provide a means of selecting and stratifying patients for immunotherapy and of predicting and monitoring response, especially when tissue sampling is insufficient or unobtainable (106).

Compared with resistance to targeted therapies, resistance to ICIs is less well defined at the molecular level. Resistance to immunotherapy is driven by both tumor-intrinsic and tumor-extrinsic mechanisms, and ctDNA-based liquid biopsy is being investigated as a minimally invasive tool to capture ICI resistance in a timely and accurate manner. Genomic features detectable through ctDNA, including alterations in *STK11* and *KEAP1*, have been associated with diminished response to PD(L)1 inhibitors (107). Noninvasive identification of these co-mutations at baseline may inform treatment selection, such as favoring chemo-immunotherapy or dual ICI therapy over ICI monotherapy. Moreover, baseline ctDNA detectability is a well-established adverse prognostic factor, associated with increased risk of recurrence in early-stage disease and progression in advanced diseases across tumor types, including thoracic malignancies. Zhang et al. demonstrated that pretreatment mean and maximum VAFs had an inverse relationship with OS after adjustment for features such as tumor burden, performance status and tumor PD-L1 score (108).

The clinical utility of ctDNA in monitoring response and detecting resistance to immunotherapy is most evident in its dynamic assessment during therapy. Various tumor-naïve and tumor-informed ctDNA assays have been evaluated for immunotherapy response assessment in late-stage NSCLC (55, 109) and SCLC (49), collectively shown that early ctDNA dynamics and ctDNA clearance serve as early indicators of therapy response. Reductions in ctDNA levels to the molecular response level have been shown to precede radiographic response and are associated with improved objective response rates, PFS, and OS. These findings are consistent across multiple assays, enabling identification of molecular responders within weeks of treatment initiation, often substantially earlier than imaging (47, 48, 110, 111). Reductions in ctDNA as early as 3 weeks after initiation of pembrolizumab-based therapies in advanced NSCLC have been associated with improved response rates and survival outcomes (111). Another study in patients with advanced NSCLC and radiographically stable disease demonstrated that ctDNA molecular responses refine response assessment and can capture differences in PFS and OS (48). In contrast, persistent or rising ctDNA levels identifies patients at high risk for primary resistance or the development of acquired resistance. In acquired resistance, serial ctDNA monitoring has revealed dynamic changes in blood TMB and neoantigen landscapes over time. Emerging data suggest that rising ctDNA levels during ICI may precede radiographic progression and reflect immune escape mechanisms, such as loss of antigen presentation or clonal selection of immune-resistant subpopulations (112).

Beyond genomic alterations, cfDNA 5-hydroxymethylcytosine (5hmC) predicts ICI response, resistance, and outcomes in advanced lung cancer by capturing immune- and epithelial-to-mesenchymal transition (EMT)-related changes (65) and may offer improved prediction of clinical response to ICIs ± chemotherapy compared with PD-L1 TPS, although limited by the small patient cohort (n=32) (84.4% vs. 46.9%; 95% CI, 67.2–94.7% vs. 29.1–65.3%; p=0.0033) (113). Similarly, in NSCLC patients receiving anti-PD-1 therapy, early changes in cfDNA tumor methylation scores within 4–10 weeks of treatment initiation strongly correlated with real-world PFS and tracked tumor burden (114), collectively highlighting the utility of methylation-based ctDNA profiling for early and longitudinal monitoring of immunotherapy response.

Although these findings are not yet routinely actionable, they provide important biological insights and a potential framework for future adaptive treatment strategies. Currently, NCT06227728 is evaluating whether ctDNA dynamics in combination with PD-L1 expression, TMB, and microsatellite instability can improve the prediction and monitoring of response to ICIs in patients with advanced cancers, including thoracic malignancies. In line with this translational shift, the field is moving from observational correlations toward prospective ctDNA-guided adaptive treatment strategies. Several clinical trials are evaluating the use of ctDNA to guide escalation or de-escalation of therapy. The MERMAID-1 (NCT0485368) and MERMAID-2 (NCT04642469) phase III trials, which both closed enrollment early, represented complementary ctDNA-guided strategies in resected NSCLC. MERMAID-1 investigated whether ctDNA MRD detection can risk stratify patients that benefit most from adjuvant durvalumab, whereas MERMAID-2 evaluated an approach in which adjuvant immunotherapy is initiated upon detection of molecular relapse after curative intent therapy. While MERMAID-1 and MERMAID-2 did not reach its primary endpoint of disease-free survival, the results are limited by a smaller sample size and limited follow up. The BR.36 trial (NCT04093167) is a multicenter, biomarker-driven phase II adaptive study enrolling patients with advanced NSCLC without actionable genomic alterations. Stage 1 of this trial identified optimal timing of ctDNA assessment as after two cycles of pembrolizumab, which was concordant with radiographic response (109). In subsequent phases, patients identified as high risk for progression based on ctDNA dynamics are being randomized to treatment intensification versus continuing immunotherapy monotherapy. Another ongoing study, the PRELUCA trial (NCT05889247) is evaluating whether patients who achieve a molecular response can safely discontinue immunotherapy prior to the conventional two years of immunotherapy, with the primary outcome as OS.

#### 2.2.3 Minimal residual disease detection and identification of molecular relapse

Minimal residual disease (MRD) assessment using liquid biopsy is an evolving field within thoracic oncology, with significant implications for early relapse, detection, and treatment personalization. Many studies demonstrate that ctDNA-based MRD positivity may precede radiographic relapse by months, suggesting earlier therapeutic intervention. Additionally, the persistence of ctDNA after curative-intent treatment, including surgical resection or chemoradiation, has been associated with worse prognosis, highlighting its potential role as a biomarker of residual disease burden and resistance (11, 18, 112). Consequently, non-invasive ctDNA analyses can guide treatment decisions by detecting MRD and emerging therapy resistance, often earlier than standard radiologic imaging (115). Assessing ctDNA at pre-defined key timepoints (landmark analysis) and tracking dynamic changes over multiple timepoints (longitudinal analysis) enables timely interception of residual disease before clinical or radiologic evidence during surveillance and treatment periods (8).

Tumor-naïve and tumor-informed mutation-based ctDNA approaches have been tested in resectable esophageal cancer (50), NSCLC (38, 116), and diffuse pleural mesothelioma (61)—through MRD detection and real-time perioperative monitoring of response to neoadjuvant and adjuvant therapies. For instance, tumor-informed bespoke ctDNA assays have demonstrated survival benefits in the setting of neoadjuvant immunotherapy (116), as well as standard adjuvant chemotherapy ± radiotherapy (24, 25) in early-stage NSCLC. By increasing the number of patient-specific variants identified from matched tumor and WBC WGS to as many as ~1,800, pre-operative ctDNA positivity predicted worse survival in patients with early-stage NSCLC adenocarcinoma in the TRACERx study (117).

In resectable thoracic cancers, ctDNA has demonstrated utility in detecting MRD and monitoring immunotherapy response in the perioperative setting. In a neoadjuvant nivolumab clinical trial in stage I to IIIA surgically resectable NSCLC (n = 14), all tumors with a major or partial pathologic response to nivolumab were molecular responders by ctDNA analysis, whereas nonresponders lacked ctDNA clearance (47). In a phase 2 trial of perioperative nivolumab or nivolumab and ipililumab combination in resectable epithelioid/biphasic diffuse pleural mesothelioma, persistent ctDNA detection at cycle 3 and pre-surgery was associated with significantly shorter PFS, while ctDNA clearance correlated with improved outcomes (HR 0.32 and 0.29, respectively) (61). Similar findings can be found in other tumor types, such as resectable gastroesophageal cancer, where undetectable ctDNA following neoadjuvant nivolumab or nivolumab-relatlimab in combination with chemotherapy and radiation therapy independent of PD-L1 expression had significantly longer recurrence-free survival (RFS) and OS. In addition, among patients who did not achieve a pathologic complete response, those with undetectable ctDNA post immunotherapy had longer RFS compared to those with detectable ctDNA (median RFS not reached versus 21.54 months; p = 0.058) (50).

From a therapeutic resistance standpoint, MRD testing may reveal the emergence of resistance clones at very low VAFs before overt disease progression. Serial ctDNA analysis allows for tracking of clonal dynamics, including the persistence, expansion, or disappearance of specific resistance-associated alterations. For example, the re-emergence of *EGFR* mutations or the appearance of bypass pathway alterations during adjuvant or consolidation therapy may signal impending resistance (18). However, ctDNA detection may be challenging in patients with low tumor burden, slowly proliferating tumors, or limited ctDNA shedding. Optimal MRD testing schedules and thresholds for clinical action, including treatment escalation or de-escalation, remain under active investigation.

## 3 CTC-based assays

### 3.1 CTCs as a complementary liquid biopsy modality

**Figure 2:**
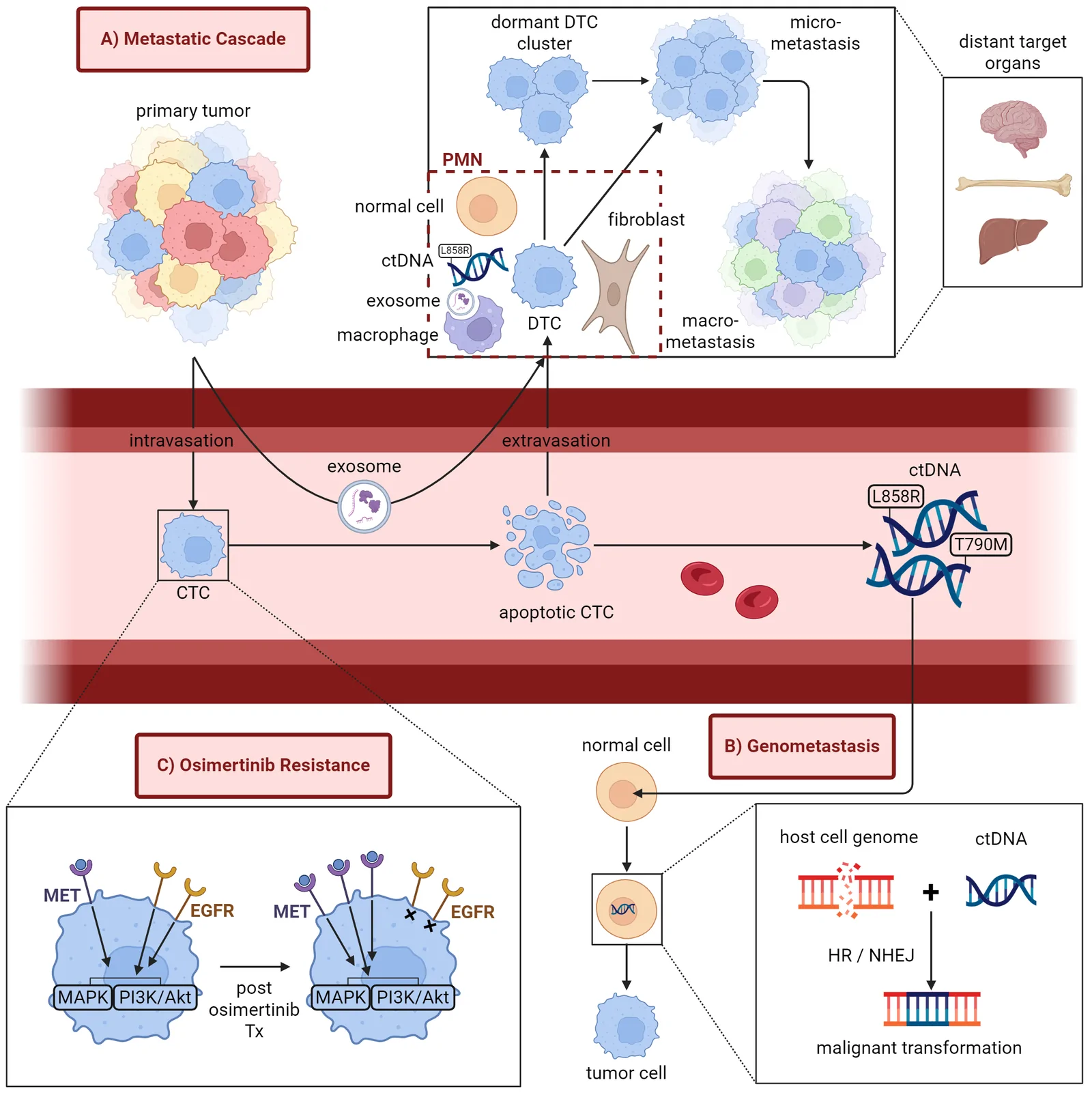
**(A)** The metastatic cascade describes the process whereby cancer cells detach from the primary tumor, migrate through the body via the circulatory system, and colonize one or more distant organs. Potential sites of metastasis can be primed by tumor-derived exosomes and circulating tumor DNA (ctDNA) to create pre-metastatic niches (PMNs), which attract circulating tumor cells (CTCs) and promote their extravasation. Following extravasation, a CTC becomes known as a disseminated tumor cell (DTC). Most CTCs die in circulation, releasing their cellular components into the blood, including fragments of ctDNA, which harbor harmful mutations. **(B)** The genometastasis theory outlines the putative role of ctDNA in metastasis. Following uptake into a host cell, ctDNA can integrate into the host cell genome through DNA damage repair pathways, such as homologous recombination (HR) or non-homologous end-joining (NHEJ), resulting in malignant transformation of the host cell. **(C)** Osimertinib resistance: Osimertinib is a medication that inhibits the activity of specific EGFR mutants, such as those with L858R and T790M mutations. Resistance to osimertinib is quite common and involves diverse mechanisms, including amplification of the *MET* gene, which leads to reactivation of cell growth and survival signaling pathways. DTC, disseminated tumor cell; PMN, pre-metastatic niches; ctDNA, circulating tumor DNA; CTC, circulating tumor cell; Tx, treatment; HR, homologous recombination; NHEJ, non-homologous end-joining. Created in https://BioRender.com.

In addition to ctDNA approaches, CTCs are a complementary liquid biopsy modality that can provide biological and functional insights into treatment response and disease evolution. First described in 1869 in the blood of a patient with metastatic disease (118), CTCs represent cancer cells that have entered the bloodstream through passive shedding or active invasion the latter driven primarily by EMT. Although rare, particularly in NSCLC (~ 1–10 cells per 10 mL of blood), CTCs circulate as either single cells or multicellular clusters and are uniquely accessible through a minimally invasive blood draw. Clusters carry greater metastatic potential (119, 120) but may exhibit a markedly shorter circulatory half-life (6–10 minutes) compared to single CTCs (25–30 minutes) in mouse models (120). Upon extravasation, these disseminated tumor cells (DTCs) may seed and colonize distant organs, spreading throughout the parenchyma of major organs (**Figure 2**) (121). However, only a small minority approximately 0.02% of DTCs ultimately generate proliferating metastatic lesions. Consistent with both the seed-and-soil hypothesis and mechanical theories of metastasis, organ-specific metastatic patterns in lung adenocarcinoma have been linked to specific molecular drivers, including alterations in *TP53*, *SMARCA4*, and *CDKN2A*, which are associated with aggressive disease phenotypes and preferential spread to sites such as the brain and bone (122–124).

### 3.2 CTC phenotypic heterogeneity and plasticity

A further layer of complexity arises from the phenotypic plasticity of CTCs, which gives rise to a heterogeneous cell population exhibiting epithelial, mesenchymal, or hybrid epithelial/mesenchymal (E/M) phenotypes. Hybrid CTCs are of particular clinical interest in a detached, circulating state, they demonstrate enhanced survival and chemoresistance, increased migratory capacity, and stem cell-like properties (125, 126). Seo et al. used an advanced imaging platform (HDSCA3.0) to identify CTC heterogeneity in SCLC patients. They identified 8 distinct CTC groups characterized by combinations of epithelial, endothelial, and mesenchymal marker expression patterns. Single-cell copy number alteration (scCNA) analysis revealed that these distinct CTC types shared similar clonal genomic alterations characteristic of SCLC. This implies that these phenotypically diverse CTCs arose due to cellular plasticity rather than from different genetic origins (127).

### 3.3 Methods for CTC isolation and detection in lung cancer

CTC analysis has not yet been widely adopted in routine clinical practice for lung cancer management, despite showing promise as a prognostic and predictive biomarker. The primary reasons for this delay, are rooted in both biological and technical challenges including; their rarity, short half-life in circulation (128), lack of reliable CTC isolation technologies and standardization, leukocyte contamination and heterogeneity (129). As a result of these limitations, advanced technologies have been developed to exploit specific immunological, molecular, and biophysical properties of CTCs. These enhanced methodologies are outlined in **Table 2** and can be categorized into cell surface markers, size–based or a combination approach, each with its own strengths and limitations (130, 131).

**Table 2:** Current and emerging technologies for CTC enrichment and identification in NSCLC.

Technology	Isolation Method	Advantages	Disadvantages
CellSearch^®^	Anti-EpCAM antibodies 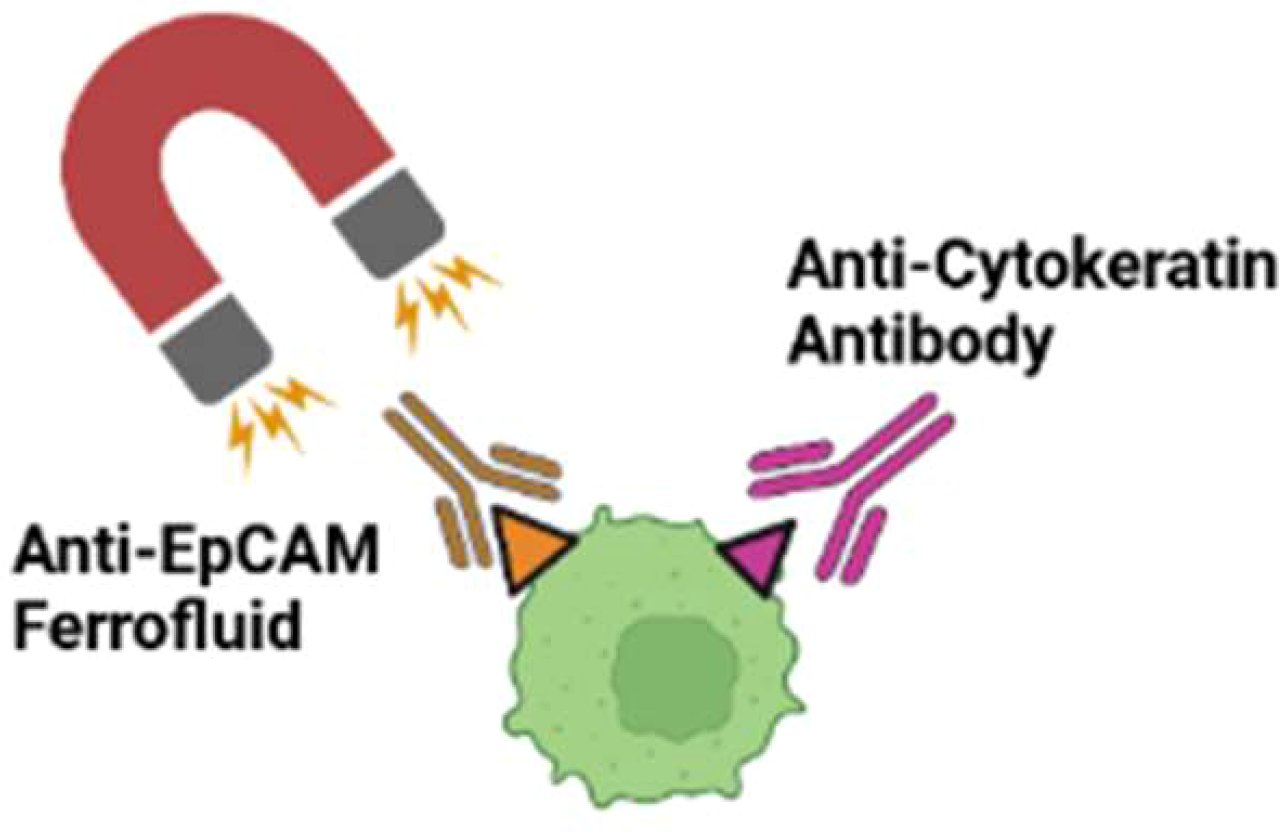	Semiautomated and FDA-approved in advanced breast, prostate and colon cancers	Only identifies EpCAM-high CTCs
ISET^®^	Size-based filtration 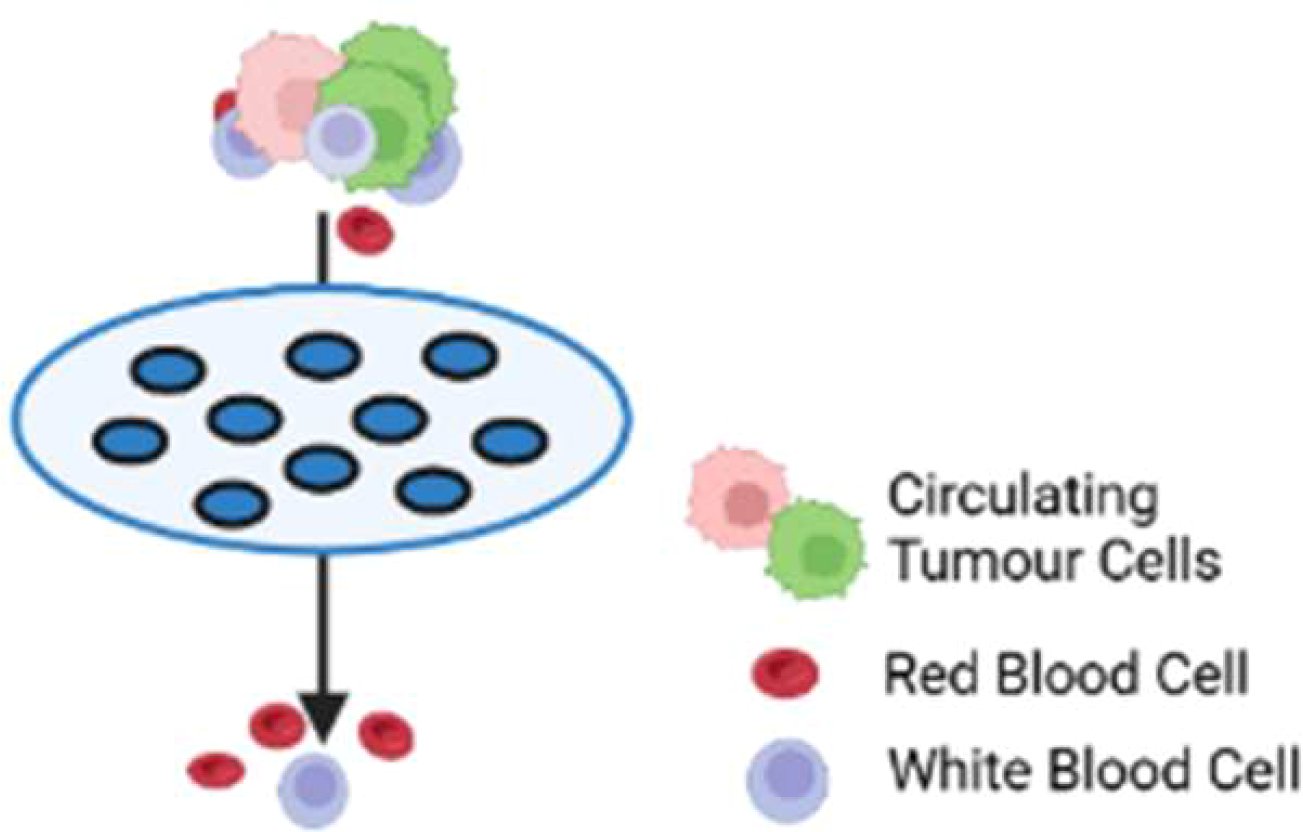	Fast Epitope Independent	CTC identification difficult and time-consuming and smaller CTCs can be lost
Parsortix^®^	Size and deformability-based microfluidics 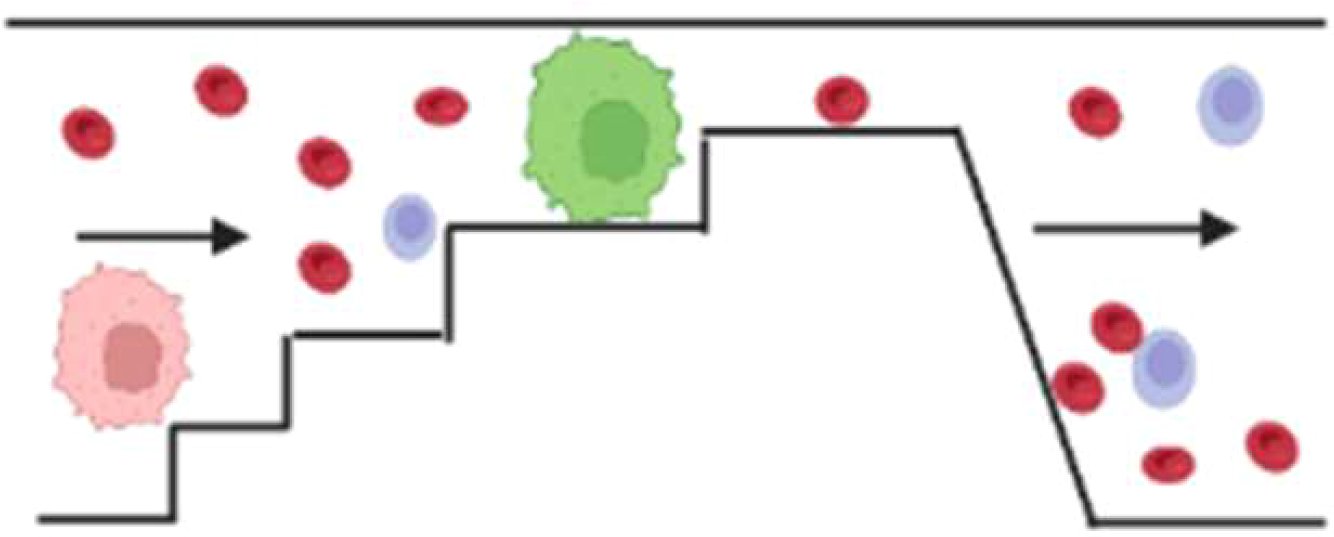	Can capture viable CTCs and clusters. Epitope Independent	Smaller CTCs can be lost. Challenge of removing leukocytes with similar size to CTCs
CanPatrol™	RNA *in situ* analysis 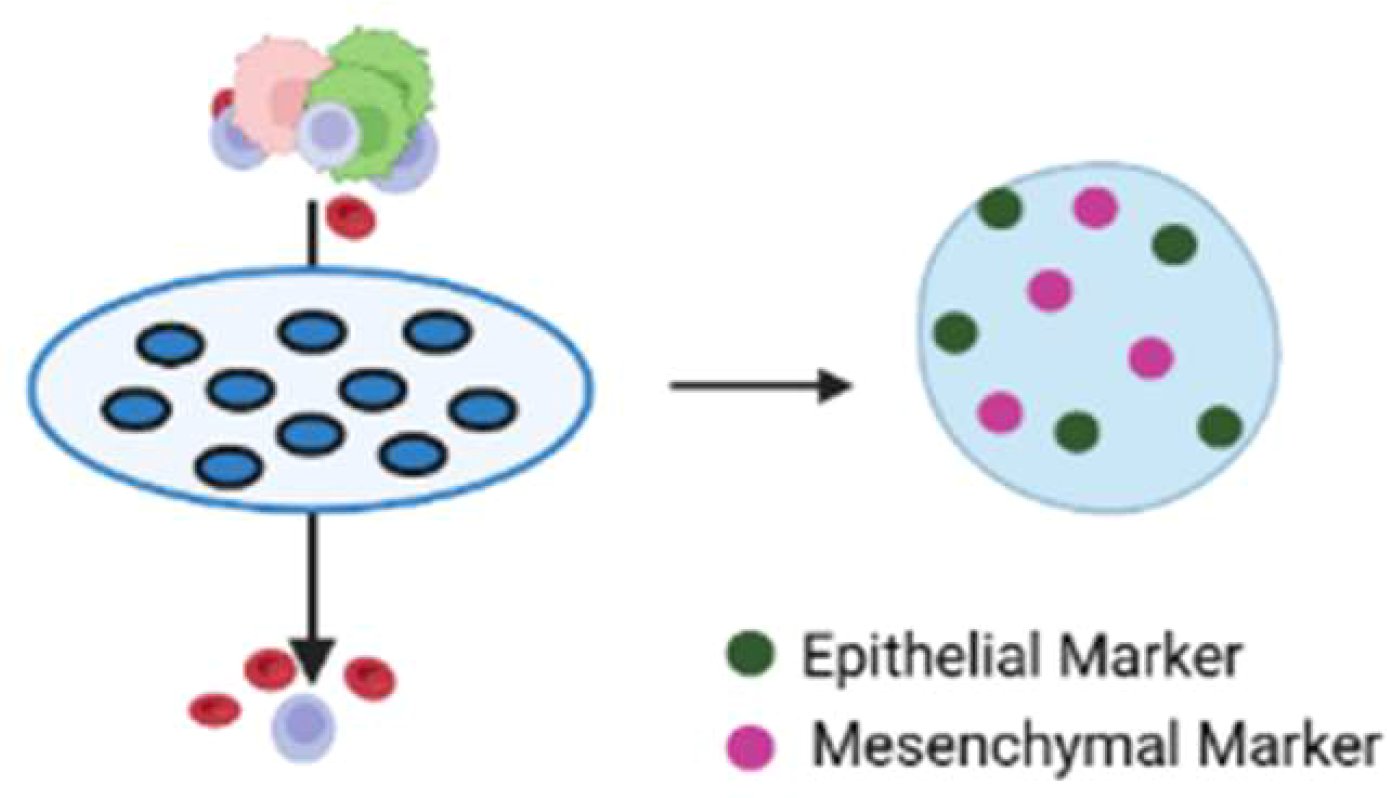	Epitope Independent	Blood characteristics such as viscosity may negatively affect analysis
Genesis	Size-based microfluidics 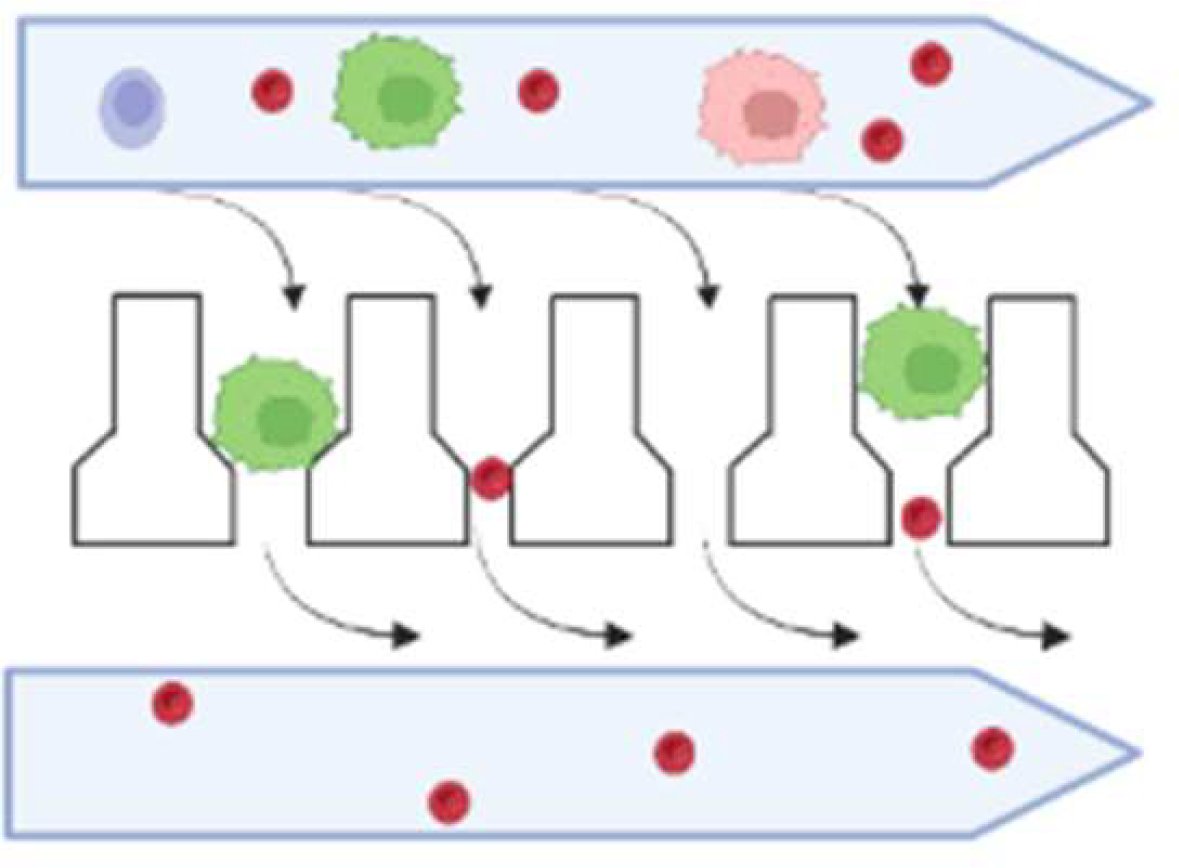	Fast and high recovery. Epitope Independent	Smaller CTCs can be lost. Challenge of removing leukocytes with similar size to CTCs

EpCAM, epithelial cell adhesion molecule; FDA, Food and Drug Administration; CTCs, circulating tumor cells; ISET®, Isolation by size of epithelial tumor cells.

Most cancers are of epithelial origin; therefore, initial technologies for CTC isolation were based on the positive selection of the Epithelial Cell Adhesion Molecule (EpCAM) antigen. EpCAM is universally expressed in epithelial tumors but not on the surface of hematopoietic cells. The CellSearch^®^ system, which relies on EpCAM, was the first FDA-approved for clinical prognostication in advanced breast, colon and prostate cancer. This system utilizes a ferrofluid containing magnetic particles coated with anti-EpCAM antibodies. The captured cells are then labelled with fluorescent reagents (DAPI, Cytokeratin (CK) and CD45) for identification and quantification of CTCs that are EpCAM+, CK+, and CD45− (132). The CellSearch^®^ system demonstrated limited sensitivity in early-stage lung cancer, although it was able to differentiate between stage I and IV disease and predict distant metastasis, especially in SCLC (133). In another study of NSCLC and SCLC patient cohorts, the CellSearch^®^ only detected CTCs in 21% of advanced NSCLC patients while the median number of CTCs for SCLC patients was 24 per patient (range 0–44.896 CTCs/7.5 mL of blood) (134). Krebs et al. detected CTCs in less than 5% of stage III patients and only 32% of stage IV patients when using a threshold of ≥1 CTC (135, 136). A key limitation of the CellSearch^®^ system is its reliance on EpCAM positivity which restricts its ability to isolate heterogenous CTCs and those that have undergone EMT, characterized by reduced epithelial and increased mesenchymal marker expression. The heterogeneous nature of NSCLC highlights that non-antigen-dependent CTC isolation technologies may be more reliable (137, 138). Consistent with this notion, CellSearch^®^ has demonstrated poor sensitivity in malignant pleural mesothelioma (MPM), with one study detecting CTCs in only 32.7% of MPM patients, likely due to MPM cells (of mesothelial origin) expressing little to no EpCAM (139).

CTCs are generally larger than most normal blood cells and less deformable. Therefore, microfiltration and microfluidic devices for CTC capture have been developed to exploit these properties. The Isolation by size of epithelial tumor cells (ISET^®^), a microfiltration device, filters blood through a membrane containing 8 µm pores, capturing the larger CTCs on the membrane while smaller cells flow through. Ilié and colleagues identified CTCs in 75% of advanced NSCLC patients (range of 2–256 CTCs/4 mL) using this system (140). Furthermore, ISET^®^ demonstrated higher CTC detection rates in NSCLC in direct comparison to CellSearch^®^ (136, 141). The Parsortix^®^ PR1 system is another microfluidic device that relies on the physical properties of CTCs, namely larger cell size and reduced deformability. It employs a separation cassette with multiple ‘steps’ to capture cells >6.5 µm. Numerous studies have reported that this system has higher recovery rates compared to CellSearch^®^ and ISET^®^ (142). Janning et al. compared the performance of the CellSearch^®^ and Parsortix^®^ in 97 patients with NSCLC (locally advanced or metastatic). The Parsortix^®^ detected ≥1 CTCs in 59 samples (61%) compared to the EpCAM-dependent CellSearch^®^ which detected CTCs in 31 samples (32%) (143). The clinical version of this technology, the Parsortix^®^ PC1 was also recently FDA-approved for CTC enrichment in metastatic breast cancer patients. Similarly, the Canpatrol™ assay utilizes size-based filtration (membrane containing 8 µm pores) followed by RNA *in situ* hybridization. The larger CTCs are captured on the membrane, then fixed and hybridized with fluorescent probes that target specific RNA transcripts including epithelial (EpCAM, Cytokeratins 8, 18, 19) and mesenchymal (Vimentin, TWIST) markers, along with CD45. CTCs are then categorized based on their RNA signal patterns (Epithelial/Mesenchymal/Hybrid CTCs). Huangfu et al. used the Canpatrol™ assay for CTC detection in 30 newly diagnosed lung cancer patients and CTC counts positively correlated with advanced stage, tumor size and metastasis (144).

The field of CTC isolation is extensive and rapidly evolving (131). New technologies focus mainly on label-independent capture, and the ability to work with live cells for downstream analysis. An example is the ‘CTC-chip’, a microfluidic device consisting of an array of microposts that can be coated with antibodies to capture CTCs (145). Kuwata et al. used this device for CTC detection in MPM. The CTC-chip was coated with anti-podoplanin antibody (a marker abundant in MPM) and the authors observed better sensitivity in CTC detection in MPM patients compared to the CellSearch^®^ (146). Another example of microfluidic CTC isolation technology is the Genesis System (Bio-Rad), which utilizes Celselect™ slides that contain microwells that capture cells in the range of 8-30 μm. The system also facilitates dual isolation of CTCs and cfDNA, as well as downstream applications such as single-cell RNA sequencing.

### 3.4 Molecular evaluation of CTCs reveals drivers of therapy resistance

Alterations in CTC counts and characteristics during therapy can indicate drug response or resistance (147). Beyond enumeration, further molecular and functional characterization of isolated CTCs is highly beneficial to determine their metastatic ability and potential for therapy resistance (148). Genomic profiling of CTCs enables detection of actionable mutations, drug-tolerant clones and and emerging resistance pathways, supporting real-time treatment adaptation (149, 150). Established approaches, such as fluorescence *in situ* hybridization (FISH) and PCR-based methods, including ddPCR allow targeted detection of genomic alterations (151), while single-cell and NGS technologies offer more comprehensive profiling of CTCs, revealing insights into intratumoral heterogeneity (152).

A study by Barbirou et al. used targeted NGS to identify somatic variants (SNVs and insertions/deletions (Indels)) in single CTCs isolated from 20 NSCLC patients, detecting mutations in key oncogenes and tumor suppressor genes, including *NF1, PTCH1, TP53, SMARCB1, SMAD4, KRAS*, and *ERBB2.* Both shared and patient-specific mutational profiles were observed, highlighting substantial intra- and inter-patient heterogeneity (153).

CTCs offer several advantages as a liquid biopsy platform for capturing tumor heterogeneity at the single-cell level, enabling identification of rare subclones that may drive resistance (154). While their prognostic value is well established, integrating molecular and phenotypic characterization of CTCs with longitudinal monitoring provides a more comprehensive understanding of treatment response and resistance across therapeutic modalities (155, 156).

#### 3.4.1 Role of CTCs in predicting chemotherapy resistance

The presence of CTCs in advanced NSCLC patients prior to therapy is considered a risk factor for poor response rates and chemoresistance (141). Furthermore, several studies have shown that changes in CTC counts throughout chemotherapy can reflect treatment response and have investigated the use of CTC enumeration as a potential biomarker predicting chemotherapy response in lung cancer patients using the CellSearch^®^ system. Muinelo-Romay et al. report that NSCLC patients tend to experience a decline in CTCs by the second cycle of chemotherapy treatment, indicating an early response to therapy, and that patients whose CTC counts increase after the first cycle are more likely to have progressive disease and poorer outcomes (157). A foundational study by Krebs et al. demonstrates that patients with fewer than 5 CTCs after the first cycle of chemotherapy have significantly longer PFS and OS than patients with ≥5 CTCs. Persistence of ≥5 CTCs after a single treatment cycle identifies patients with early treatment failure, with modulation of CTC counts occurring within weeks of therapy and, in some cases, preceding radiographic assessment, supporting the potential utility of on-treatment CTC dynamics as an early pharmacodynamic marker of chemotherapy response (135). Normanno et al. observed that a reduction in CTC count of >89% after one cycle of chemotherapy was associated with a markedly lower risk of death, whereas baseline CTC numbers alone did not predict outcomes (158). Similarly, Hiltermann et al. reported that decreases in CTC counts after chemotherapy were more strongly associated with OS than disease stage or radiologic response. Early reductions in CTC numbers also correlate with tumor shrinkage and longer PFS, whereas persistently high counts signal treatment resistance (159). In a phase I clinical trial, detectable CTCs after the first chemotherapy cycle could identify non-responders in extensive-stage SCLC, allowing for potential early treatment modification (160). Collectively, these studies suggest that serial CTC monitoring, rather than single baseline measurements, may provide a minimally invasive strategy to identify chemoresistance early, refine prognosis, and inform treatment decisions in both clinical trials and routine practice.

While some limited studies have identified the presence of CTC clusters as a predictor of chemotherapy response in NSCLC (161), CTC dynamics differ between histologies, with SCLC patients often presenting with more frequent CTC clusters and higher baseline CTC counts. Functional studies suggest that CTC clusters are enriched for stem-like cells, displaying enhanced cell-cell adhesion and paracrine signaling that promotes evasion of cytotoxic agents (162). Thus, increased CTC counts and increased frequency of CTC clusters may mediate collective CTC survival under cytotoxic stress and enhance chemoresistance in SCLC. Klameth et al. demonstrated that CTC cell lines established from SCLC CTCs spontaneously formed clusters in culture with dense outer layers and necrotic or hypoxic cores, demonstrating resistance to cisplatin, etoposide, topotecan, and epirubicin, compared to dispersed CTCs (163).

Beyond enumeration, phenotypic and molecular characterization of CTCs may be a useful tool for monitoring the response to chemotherapy. The presence of CTCs with an EMT phenotype has been correlated with progression, recurrence, and resistance to therapy. A study by Togo et al. provides evidence for this in NSCLC, demonstrating that patients with Vim+ CTCs at baseline had a poorer response to chemotherapy, and that the presence of total CTCs and Vim+ CTCs correlated with significantly shorter PFS than EpCAM+ CTCs (164). Comparative profiling of CTCs with paired primary and progressing tumor samples demonstrated that CTCs capture treatment-induced tumor heterogeneity, including the emergence of new somatic alterations associated with chemotherapy resistance in NSCLC (165). In this study by Chang et al., CTCs from several patients showed greater mutational overlap with progressive tumors than with primary tumors, indicating that CTCs may reflect resistant subclones selected under chemotherapy pressure. Notably, mutations affecting cell-cycle regulation, DNA damage response, and drug transport pathways were recurrently detected in CTCs from patients with platinum-refractory disease, aligning with established mechanisms of resistance to cytotoxic treatment (165). Broader measures of genomic instability in CTCs have emerged as important markers of chemotherapy response. A CNA-based predictive test applied to baseline CTCs, developed by Carter et al., can distinguish chemosensitive from chemorefractory disease in 83% of SCLC patients and predicts PFS; persistent or expanding unstable CTC populations reflect emerging resistance. Furthermore, CNA profiles at relapse did not revert in initially chemosensitive patients, suggesting that distinct genetic mechanisms drive intrinsic and acquired chemoresistance in SCLC (166). In summary, serial CTC monitoring represents a promising minimally invasive tool for assessing chemotherapy efficacy and emerging evidence indicates that phenotypic and molecular interrogation of CTCs may improve prediction of chemoresistance beyond enumeration alone.

#### 3.4.2 CTCs as dynamic indicators of immunoediting and immunotherapy response

Several studies in advanced NSCLC demonstrate that higher CTC counts at baseline are associated with poor treatment response and survival outcomes, suggesting that circulating tumor burden reflects underlying disease aggressiveness and immune resistance (167, 168). Importantly, changes in CTC counts during treatment appear to be more informative than baseline measurements alone. Early declines in CTC numbers following initiation of nivolumab or pembrolizumab correlate with radiographic response and prolonged survival, whereas persistently elevated or rising CTC counts are associated with primary resistance and early disease progression (143, 167).

Assessing PD-L1 expression in CTCs has been extensively investigated to refine patient selection and monitor responses to ICIs. While some studies report concordant classification of PD-L1 expression between NSCLC CTCs and tumor tissue (140), many studies report poor concordance, with CTCs being more frequently PD-L1 positive when compared to tissue (143, 167). These differences likely reflect spatial heterogeneity and therapy-induced immune adaptation, as CTCs preferentially originate from metastatic tumor subclones, whereby exposure to systemic inflammatory signals or prior treatment selectively enriches for PD-L1–expressing, immune-evasive cells in the circulation (169). The persistence of PD-L1–positive CTCs during ICI therapy has been associated with inferior outcomes. Guibert et al. demonstrate that NSCLC patients with detectable PD-L1 expressing CTCs prior to nivolumab treatment have significantly shorter PFS and OS, and that persistence of PD-L1 positive CTCs during therapy is associated with treatment failure (167). Furthermore, increasing PD-L1 expression on CTCs at the time of disease progression, in patients receiving PD-1/PD-L1 inhibitors, suggests adaptive immune escape under therapeutic pressure (143). EMT-related pathways can induce PD-L1 expression and phenotypic characterization of CTCs has revealed that EMT and hybrid EMT states are increasingly enriched in patients who do not attain benefit from ICIs (170). Importantly, PD-L1 expression on CTCs is closely linked to EMT and stem-like phenotypes. EMT-associated CTCs frequently display elevated PD-L1 expression and enhanced resistance to immune-mediated clearance (168, 171). These immune-evasive phenotypes are associated with poor survival outcomes and may represent a circulating reservoir of ICI-resistant tumor cells, reinforcing the concept that CTCs are not merely passive biomarkers but active participants in immune escape.

In contrast to NSCLC, SCLC exhibits low and heterogeneous PD-L1 expression, and the predictive value of tumor PD-L1 remains controversial (172). Consequently, studies assessing the role of SCLC CTCs and PD-L1+ CTCs in monitoring response to ICIs are limited. However, a recent study phenotypically characterizing SCLC CTCs has identified biologically aggressive subpopulations, with PD-L1 expression frequently co-occurring other markers linked to immune evasion and poor survival (173). Furthermore, the interplay between CTC phenotypes and circulating immune cells may influence immunotherapy outcomes. In treatment-naïve extensive-stage SCLC, patients with higher numbers of PD-L1+ CTCs together with elevated circulating CD8+PD-1+ T cells demonstrated a survival advantage following frontline ICI therapy, suggesting that CTC–immune cell interactions could offer predictive value beyond PD-L1 expression alone (174).

Beyond ICIs, emerging immunotherapeutic strategies in SCLC, particularly bispecific T-cell engagers (BiTEs) targeting delta-like ligand 3 (DLL3), introduce additional opportunities for CTC-based biomarker development. DLL3 is highly expressed on SCLC tumor cells but largely absent from normal adult tissues, making it an attractive therapeutic target. Recent studies evaluating DLL3-targeted BiTEs, such as tarlatamab, highlight the importance of accurately assessing DLL3 expression for predicting treatment response (175). In this context, CTCs offer a minimally invasive platform to longitudinally monitor DLL3 expression and tumor heterogeneity. Early evidence suggests that DLL3 expression can be detected and quantified on CTCs, with dynamic changes during therapy potentially reflecting emerging resistance mechanisms (176, 177). Although clinical data remain limited, integrating DLL3 assessment on CTCs with other circulating biomarkers may improve patient stratification and enable real-time detection of resistance to BiTEs. Emerging evidence indicates that interactions between CTCs and circulating immune cells influence tumor cell survival and may modulate responses to immunotherapy. CTCs frequently form aggregates with immune cells such as neutrophils, which can protect tumor cells from immune clearance and promote metastasis (178). Moreover, comprehensive blood biomarker studies demonstrate that early decreases in CTC counts alongside favorable immune cell profiles are associated with improved outcomes for patients receiving ICIs, suggesting that the interplay between immune contexture and CTC dynamics may inform response to immune checkpoint blockade (179). While CTC enumeration remains the most promising biomarker during ICI treatment, emerging data suggest that additional layers of CTC characterization will be necessary to improve the prediction of response and resistance to immune checkpoint blockade.

#### 3.4.3 CTCs capture molecular evolution under the selective pressure of targeted therapies

Given the genomically mediated resistance mechanisms in oncogene-driven lung cancer, molecular and genetic characterization of CTCs is particularly important for monitoring responses to targeted therapies. Several studies have shown that baseline CTC enumeration is prognostic among patients receiving TKIs. In EGFR-mutant NSCLC, higher baseline CTC counts are associated with treatment failure and shorter PFS and OS in patients treated with first- and second-generation EGFR TKIs (180, 181). Unsurprisingly, changes in CTC numbers during therapy appear to provide greater clinical value than baseline measurements alone. Yang et al. demonstrate that increases in CTC counts following initiation of EGFR-targeted therapy (erlotinib or gefitinib) in NSCLC patients is associated with early treatment failure and poor survival outcomes (180).

An early proof-of-concept study by Maheswaran et al. demonstrated that *EGFR*-activating mutations and the resistance-associated T790M mutation could be detected in CTCs from NSCLC patients, highlighting the feasibility of using CTCs to track molecular evolution under TKI pressure (182). Subsequent studies have shown that detection of T790M-positive CTCs during EGFR TKI treatment is associated with disease progression and resistance, often preceding radiographic relapse (183). The ability of CTCs to capture spatial heterogeneity and therapy-selected resistant subclones makes them particularly valuable for monitoring molecular evolution and resistance. A study by Ni et al. demonstrated that individual CTCs capture clinically actionable SNVs and CNAs that closely mirror metastatic rather than primary tumors in lung adenocarcinoma patients. Importantly, CTCs revealed enrichment of resistance-associated alterations, including *EGFR* mutations and *PIK3CA* variants linked to EGFR TKI resistance, as well as concurrent *TP53* and *RB1* mutations that preceded histologically confirmed adenocarcinoma-to-SCLC transformation (184).

CTC-based molecular profiling can capture heterogeneity and resistance mechanisms that may be missed by single-site and single-timepoint tumor biopsies. A study by Pailler et al. demonstrated no significant association between baseline numbers of *ALK*-rearranged or *ALK*-copy number gain (CNG) CTCs and survival outcomes. However, they observed a significant association between the decrease in *ALK*-CNG CTC numbers with crizotinib response and improved PFS (150). Interestingly, Pailler et al. have also demonstrated discordant *ALK* rearrangement status between primary tumors and CTCs in ALK-positive NSCLC, with *ALK*-positive CTCs persisting during crizotinib therapy and increasing at progression, suggesting selective survival of resistant subclones in circulation (185). Interestingly, this study also identified a consistent mesenchymal phenotype in *ALK*-rearranged NSCLC CTCs, regardless of tumor tissue expression of epithelial and mesenchymal markers, suggesting that ALK-driven signaling may promote EMT and selective dissemination of mesenchymal CTCs in ALK-positive NSCLC, with these invasive subclones persisting during ALK inhibition and potentially driving metastasis and treatment resistance (185). Taken together, these findings illustrate that CTC sequencing can provide early insight into clonal selection, resistance evolution, and lineage plasticity during targeted therapy, supporting its potential utility for guiding treatment adaptation when repeat tissue biopsy is not feasible.

#### 3.4.4 Cellular residual disease

Approximately 30-50% of NSCLC patients will relapse even after radical surgery, and this is attributable to post-operative MRD (186). MRD in the context of ctDNA has been extensively evaluated in lung cancer (187–189). However, evaluating CTCs as a monitoring tool for MRD (called CRD or cellular residual disease) could also be beneficial as they are live cells that can provide a more holistic view of the tumor. CTCs have some advantages over ctDNA, including real-time monitoring of the tumor’s evolving landscape and valuable biological information (DNA, RNA, proteins). Zhang et al. evaluated the clinical efficacy of monitoring peri-operative and follow-up CTC levels to predict recurrence in early-stage lung adenocarcinoma (190). They demonstrated that longitudinal monitoring of CTCs accurately predicted recurrence (AUC of 0.9786) and that CTC detection preceded radiological recurrence by a median of 183 days. Numerous other studies have reported similar findings (191, 192). Furthermore, CTCs provide increased confidence that identified multi-omics alterations are tumor-derived compared to cfDNA, which can be influenced by other factors such as clonal haematopoiesis. Serafini et al. describe how a synergistic approach combining sensitivity of ctDNA assays while harnessing the potential of CTCs (to provide insights on tumor biology) would transform residual disease monitoring in breast cancer (193). Further studies such as these investigating CRD and MRD, are warranted in lung cancer.

## 4 Multimodal liquid biopsy approaches

ctDNA and CTCs are the most widely studied liquid biopsy analytes, yet each provides distinct and complementary information on tumor biology as well as treatment response and resistance. ctDNA offers high analytical sensitivity for detecting tumor-derived genomic alterations and enables real-time monitoring of clonal evolution. In contrast, CTCs retain cellular and phenotypic context, allowing transcriptomic and proteomic characterization despite technical challenges and limitations related to low abundance, particularly in early-stage malignancies. Together, integrated ctDNA and CTC analysis offers a synergistic framework to overcome key limitations of single-analyte approaches and enhance the clinical utility of liquid biopsy (6, 7, 194).

Although studies using multi-analyte liquid biopsy approaches remain limited by technical and logistical constraints, accumulating evidence from ongoing studies and clinical trials supports their complementary value for diagnosis, prognosis, and treatment selection. In advanced NSCLC treated with TKIs, combined mutation-based ctDNA and CTC analyses targeting EGFR or MET enable non-invasive, real-time detection of emerging resistance mutations under treatment pressure, informing next-line therapy decisions (183, 195, 196). In an early study on the *EGFR* T790M resistance mutation, combined CTC and ctDNA analysis detected the mutation in ~47–50% of patients, with 57–74% concordance to tissue biopsy, and identified resistance in 35% of cases with negative or indeterminate tissue results (183). Similarly, Ntzifa et al. analyzed dynamic changes in ctDNA and CTC methylation, with or without mutation profiling, in NSCLC patients before treatment and at progression on the third-generation EGFR TKI osimertinib (197, 198). Methylation of selected genes in plasma cfDNA and paired CTCs significantly increased at progression (p = 0.031), with low concordance between analytes (197). Combined epigenetic and genetic profiling revealed complementary resistance mechanisms and clinically actionable alterations—including baseline T790M mutations in CTCs, *PIK3CA* mutations in cfDNA, *MET* and *HER2* amplifications, increased vimentin- and PD-L1-positive CTCs at progression, and AXL/PIM-1 expression during treatment—demonstrating that serial multi-analyte liquid biopsy can capture tumor heterogeneity and inform subsequent therapy decisions (198).

Beyond the TKI setting, in advanced NSCLC patients treated with ICIs, combined ctDNA and CTC analyses provide strong prognostic and predictive value, with baseline levels and early dynamics identifying high-risk patients with poor survival and low-risk subgroups most likely to derive durable benefit from immunotherapy (199, 200). Consistent with these findings, the complementary prognostic value of combined ctDNA and CTC analyses has also been demonstrated in advanced NSCLC populations with diverse treatment histories, including chemotherapy, radiotherapy, immunotherapy, targeted therapy, and combination regimens (201, 202). Kapeleris et al. investigated CTC and cfDNA levels from advanced stage NSCLC patients and compared it to their time to progression (201). They observed that CTC cluster counts and cfDNA levels were associated with shorter PFS. Notably, Kong et al. showed substantial genomic heterogeneity across ctDNA and CTCs, with greater concordance to metastatic than primary lesions, and dynamic evolution from treatment to progression, underscoring their complementary value for monitoring metastatic evolution in lung and breast cancer (202). Although most multi-analyte liquid biopsy studies in thoracic malignancies focus on advanced NSCLC, preoperative analysis of *BRAF*, *KRAS*, *EGFR*, and *PIK3CA* in early-stage NSCLC showed that combined ctDNA and CTC assessment increased mutation detection from 24.5–38.8% to ~53%, with any mutation in either analyte predicting higher relapse risk, supporting integrated liquid biopsy as a pre-surgical prognostic tool (203). Beyond NSCLC, cfDNA sequencing detected tumor-derived alterations in 94% of limited-stage SCLC (rising to 95% with parallel CTC analysis) and 100% of extensive-stage patients, with cfDNA copy-number metrics and CTC counts correlating with disease stage and OS, and identifying actionable targets identified in over 50% of cases (204).

Although most studies support the complementary value of combined multi-analyte liquid biopsy analyses, a limited number have reported the superiority of ctDNA over CTCs, or vice versa. In EGFR-mutant NSCLC patients treated with osimertinib, early decreases and clearance of ctDNA after one treatment cycle were strongly associated with significantly longer PFS and OS, while detectable baseline ctDNA independently predicted shorter PFS (HR 3.0, p=0.009); in contrast, no predictive value was observed for CTC counts (205). Similarly, prior to chemotherapy initiation in advanced NSCLC, baseline cfDNA levels were independently associated with significantly worse OS and markers of tumor aggressiveness (i.e. high tumor metabolic activity related to metastatic burden), whereas inverse or no significant associations were observed with CTC analyses alone (206, 207). By contrast, results from the ALCINA 1 trial in advanced NSCLC showed that ctDNA mutations (detected in 37% of patients) were not associated with PFS or OS, whereas high PD-L1^+^ small EV (sEV) levels and the presence of CTCs were significantly associated with poorer outcomes. Notably, patients with both detectable CTCs and high PD-L1^+^ sEV concentrations had a markedly worse prognosis (OS HR = 7.65, *p* < 0.001), highlighting the strong prognostic value of combined CTC and EV analysis (208). A separate study combining ctDNA, CTC, and EV analyses in advanced NSCLC demonstrated that EpCAM^high^ CTCs (cut-off ≥2), elevated tumor-derived EVs (cut-off ≥18), and high-allele frequency ctDNA (≥10%) were each associated with poor OS, whereas EpCAM^low^ CTCs were not. Importantly, patients harboring two or more unfavorable biomarkers had significantly worse OS, supporting the prognostic utility of a single-tube, multi-analyte liquid biopsy approach (209). These findings highlight the added prognostic value of integrating multiple circulating analytes beyond ctDNA and CTCs.

The limitations of repeated tissue biopsies and imaging hinder the timely and accurate capture of the dynamic, continuous interactions among tumor, immune, and stromal cell populations within the tumor microenvironment (TME), which play a substantial role in treatment resistance, particularly under immunotherapy pressure (112, 210). Investigating these interactions may elucidate the biology of tumor-host dynamics underlying emerging resistance, serve as an early indicator of treatment response, and enable timely interception of therapy resistance. Longitudinal, multimodal analyses of reliable, non-invasive biomarkers that reflect evolving tumor-immune-stromal remodeling in the peripheral circulation hold promise for addressing this gap (48, 112, 210–212). For instance, integration of ctDNA kinetics with peripheral immune biomarkers may enhance predictive accuracy of treatment response in the context of immunotherapy. Peripheral blood cell indices, such as the neutrophil-lymphocyte ratio and eosinophil counts, have demonstrated improved prediction of treatment response when combined with ctDNA dynamics (211). In addition, longitudinal peripheral T-cell receptor (TCR) sequencing in metastatic NSCLC patients treated with immunotherapy containing regimens revealed significant clonotypic TCR expansions and regressions occurring a median of 18 weeks prior to clinical presentation of immune-related adverse events (irAEs) in 15 of the 17 patients who developed an irAE (48). Patients who developed irAEs had higher baseline expression of proinflammatory proteins and increased on-treatment expression of inflammatory cytokines. These findings highlight the potential of combined ctDNA and immune monitoring for predicting and tracking irAEs, in addition to capturing immunotherapy resistance. Moreover, although cancer-associated fibroblasts (CAFs) within the TME are well recognized as a supportive “soil” niche for metastatic “seeds”, i.e. cancer cells (213), the role of circulating CAFs (cCAFs) as predictive and prognostic liquid biopsy biomarkers remains less explored, especially in thoracic malignancies (210, 214, 215). A pan-cancer study including lung cancer patients (7 of 52 patients) demonstrated that elevated levels of cCAFs, either alone or in clusters with CTCs, were associated with poor prognosis and probability of shorter OS in metastatic cancer patients receiving chemotherapy, whereas CTCs alone were not prognostic (214). Similarly, higher cCAF expression levels were correlated with advanced disease stage in 25 lung cancer patients (stage III–IV vs. stage I–II; p = 0.014) (215). In both studies, α-smooth muscle actin (α-SMA)-positive cCAFs were not detected in healthy donor blood, suggesting a more pronounced role for cCAFs in the metastatic setting and during cancer progression (214, 215). Collectively, these findings underscore the added value of TME-associated liquid biopsy approaches for predicting patient outcomes, capturing resistance, monitoring treatment responses, as well as treatment-related adverse events.

In addition to combining multiple liquid biopsy analytes, integrating these with artificial intelligence-assisted analytics, including radiomics and multi-omic machine learning models, allows multimodal liquid biopsy platforms to identify complex resistance patterns that would remain undetected with single biomarkers alone (216, 217). For example, in 418 patients with locally advanced NSCLC treated with chemoradiotherapy, mid-treatment ctDNA levels were prognostic for PFS, and their integration with pre-treatment tumor features and radiomics further improved risk stratification beyond any single modality, supporting response-adapted treatment strategies (218). Recent advances in machine learning-assisted proteomic analyses enable high-throughput profiling of TME-associated proteins (up to ~11,000 proteins) using relatively low plasma volumes (~1-130 µL), holding great potential to capture tumor-host interactions more effectively than tumor tissue-based markers alone. PROphet is an aptamer-based plasma proteomics platform integrated with a machine learning method to complement standard PD-L1-guided patient stratification for ICI treatment by predicting clinical benefit based on resistance-associated protein scores (219, 220). In the PROPHETIC (NCT04056247) trial cohort, the platform stratified patients with metastatic NSCLC who benefited most from ICI and chemotherapy combination therapy compared to ICI monotherapy in the PD-L1 high subgroups (220). Predicted OS outcomes for metastatic NSCLC patients treated with ICI, chemotherapy, or their combination showed high concordance with real-world data (R^2^ = 0.98, P < 0.001). Collectively, these advances position multimodal liquid biopsy as a powerful tool for dynamic, real-time assessment of resistance biology, tumor monitoring, and guiding precision treatment strategies.

## 5 Clinical barriers & future directions

Despite its growing promise in elucidating tumor evolution, clonal heterogeneity, and mechanisms of therapy resistance, liquid biopsy has not yet been fully integrated into routine clinical decision-making. While longitudinal assessment of ctDNA, CTCs, and other circulating analytes offers unique insight into tumor dynamics under selective therapeutic pressure—supporting concepts such as real-time monitoring, adaptive therapy, and early interception of resistance—several analytical, clinical, and logistical barriers remain. Particularly relevant to ctDNA assays, assay design, depth of sequencing, bioinformatic pipelines, and limits of detection vary significantly across platforms, complicating cross-trial comparisons and clinical interpretation (12, 221). In clinical practice, ctDNA assay selection is often physician-dependent, and repeat liquid biopsy is typically performed at time of recurrence or progression. Key questions that have not been fully addressed include the optimal timing and frequency of testing, and standardized thresholds for action that balance potential benefits of early intervention and risks of overtreatment.

A major challenge relates to the trade-offs between assay sensitivity and breadth, which must be carefully aligned with the intended clinical application. Hybrid capture-based targeted sequencing panels enable broader genomic profiling and discovery of *de novo* or unexpected resistance mechanisms but often lack the analytical sensitivity required for detecting low-frequency variants in early-stage or low tumor burden disease. In contrast, targeted mutation-based PCR assays provide superior sensitivity for longitudinal tracking and MRD detection when the mutation of interest is known, yet may miss emergent resistance alterations outside the predefined target space. As a result, a “negative” liquid biopsy—particularly in early-stage disease or during deep treatment responses—does not exclude the presence of residual disease or resistant clones. Sensitivity limitations are especially pronounced for CTC detection and ctDNA quantification in low-burden settings, underscoring the need for improved technologies and standardized reporting frameworks, as recently proposed by the European Liquid Biopsy Society (ELBS)-led expert consensus for cfDNA-based NGS reporting (222).

Beyond technical considerations, the clinical significance of low-level ctDNA or CTC positivity, particularly in the absence of radiographic progression remains uncertain. Although rising ctDNA levels may signal emerging resistance and precede imaging-detectable relapse or resistance, it is not yet well-established whether therapeutic intervention at the time of molecular progression improves outcomes compared with continuation of therapy until radiographic progression. Additionally, frequent testing may increase patient anxiety and healthcare costs without proven clinical benefit. To address these challenges, ongoing clinical trials are testing the value of ctDNA molecular response in treatment optimization for patients with thoracic malignancies.

Future directions are likely to center on multimodal liquid biopsy strategies that integrate multiple circulating analytes—such as ctDNA, cfDNA fragmentomic or epigenomic features, CTCs, EVs, and circulating tumor-associated proteins—together with artificial intelligence-assisted analytical frameworks. Such approaches may overcome the limitations of single-analyte assays by providing a more comprehensive view of tumor biology, enhancing sensitivity for resistance detection, and improving risk stratification for adaptive therapeutic strategies. Ultimately, rigorously designed clinical trials incorporating standardized liquid biopsy methodologies, predefined molecular endpoints, and integrated multimodal analyses will be essential to define the clinical utility of liquid biopsy-guided surveillance, treatment escalation, and de-escalation strategies across the cancer care continuum.
